# Identification of Flowering-Related Genes Responsible for Differences in Bolting Time between Two Radish Inbred Lines

**DOI:** 10.3389/fpls.2016.01844

**Published:** 2016-12-09

**Authors:** Won Yong Jung, Hyun Ji Park, Areum Lee, Sang Sook Lee, Youn-Sung Kim, Hye Sun Cho

**Affiliations:** ^1^Plant Systems Engineering Research Center, Korea Research Institute of Bioscience and BiotechnologyDaejeon, Korea; ^2^Biosystems and Bioengineering Program, University of Science and TechnologyDaejeon, South Korea; ^3^Department of Biotechnology, NongHyup SeedAnseong, South Korea

**Keywords:** flowering time-related gene, late bolting, radish (*Raphanus sativus* L.*)*, RNA sequencing, vernalization

## Abstract

Late bolting after cold exposure is an economically important characteristic of radish (*Raphanus sativus* L.), an important Brassicaceae root vegetable crop. However, little information is available regarding the genes and pathways that govern flowering time in this species. We performed high-throughput RNA sequencing analysis to elucidate the molecular mechanisms that determine the differences in flowering times between two radish lines, NH-JS1 (late bolting) and NH-JS2 (early bolting). In total, 71,188 unigenes were identified by reference-guided assembly, of which 309, 788, and 980 genes were differentially expressed between the two inbred lines after 0, 15, and 35 days of vernalization, respectively. Among these genes, 218 homologs of *Arabidopsis* flowering-time (Ft) genes were identified in the radish, and 49 of these genes were differentially expressed between the two radish lines in the presence or absence of vernalization treatment. Most of the Ft genes up-regulated in NH-JS1 vs. NH-JS2 were repressors of flowering, such as *RsFLC*, consistent with the late-bolting phenotype of NH-JS1. Although, the functions of genes down-regulated in NH-JS1 were less consistent with late-bolting characteristics than the up-regulated Ft genes, several Ft enhancer genes, including *RsSOC1*, a key floral integrator, showed an appropriate expression to the late-bolting phenotype. In addition, the patterns of gene expression related to the vernalization pathway closely corresponded with the different bolting times of the two inbred lines. These results suggest that the vernalization pathway is conserved between radish and *Arabidopsis*.

## Introduction

Radish (*Raphanus sativus* L.), an annual plant belonging to the Brassicaceae family, is a familiar root vegetable crop around the world, especially in eastern Asia. The main edible portion of the plant is the fleshy taproot, which has an upper part originating from the hypocotyl and a lower part consisting of true root tissue. The taproot exhibits huge variation in terms of skin color and shape (Tsuro et al., 2008; Zaki Hem and Yokoi, 2012). Some cultivars of radish have been exploited as oilseed crops, silique vegetables, and leafy vegetables. The taproot is an excellent source of carbohydrates and dietary fiber, high levels of glucosinolates and secondary metabolites for human beings: consequently, the properties of the root are the primary determinants of the crop's economic value (Nakamura et al., 2001; Wang et al., 2002). Comparative genomics revealed that the *Brassicaceae* species exhibit complex genomic syntenies (Panjabi et al., 2008; Li et al., 2011), indicating that a wide range of whole-genome rearrangements occurred during or after species divergence. By contrast, genome syntenies are well conserved in Poaceae (International Brachypodium, 2010) and Solanaceae crops (Tomato Genome, 2012). The genome of the radish is relatively small and diploid (2*n* = 2*x* = 18), and analysis of genetic map construction and comparative mapping based on EST-SSR (Shirasawa et al., 2011) or EST-SNP markers (Li et al., 2011) revealed that it is closely related to *Brassica rapa*. Two whole draft genome sequences of radish were recently reported: the 402 Mb sequence of Japanese cultivar “Aokubi” and 510 Mb sequence of Korean cultivar “WK10039” (Kitashiba et al., 2014; Mun et al., 2015). However, establishment of the more accurate chromosome pseudomolecules by clone-end and BAC-end sequences remains to be accomplished.

In flowering plants, proper timing of the transition from vegetative to reproductive development is important to ensure reproductive success and seed production (Srikanth and Schmid, 2011); accordingly, this trait has considerable economic value. Regulation of Ft is controlled by a diverse range of environmental and internal signals (Koornneef et al., 1998). Environmental signals, which are strongly influenced by season, day length, and temperature adaptation, are integrated with endogenous signals such as developmental stage and age (Zhai et al., 2015). In the model plant *Arabidopsis*, the majority of key Ft genes have been identified and characterized through genetic and molecular analyses. These genes can be categorized into five main flowering pathways: the photoperiod, vernalization, gibberellin, autonomous, and endogenous pathways (Putterill et al., 2004; Fornara et al., 2010; Srikanth and Schmid, 2011). However, these genetically established pathways are not rigorously distinguished. Instead, a growing body of evidence indicates that extensive crosstalk occurs between the pathways, and that the resultant signals are ultimately integrated by a small number of common target genes (central floral pathway integrator genes) that quantitatively regulate the development of shoot apical meristem. Consequently, control of Ft is plastic and diverse (Simpson and Dean, 2002). Functional genetic analysis in *Arabidopsis* revealed that the *FLOWERING LOCUS T* (*FT*), *SUPPRESSOR OF OVEREXPRESSION OF CONSTANS1* (*SOC1*), and *LEAFY* (*LFY*) genes are central floral integrators (Blazquez et al., 1998; Nilsson et al., 1998; Samach et al., 2000). FT has been known for florigen, a mobile signal that transfers the flowering induction signal from leaves into shoot apical meristems through the phloem (Tamaki et al., 2007; Turck et al., 2008), and this protein is highly conserved in most flowering plants (Andrés and Coupland, 2012). SOC1 encodes a MADS box protein that acts as a floral activator to control floral patterning and floral meristem, as well as Ft (Liu et al., 2007, 2009; Melzer et al., 2008). LFY, a unique plant transcription factor that also contains a MADS box, is indispensable for determining the identity of male and female reproductive organs during flower development (Weigel et al., 1992). Together, these key integrator genes interpret signals from a global network of multiple flowering pathways, and their expression levels precisely modulate the expression of floral meristem-specific genes and ultimately determine the exact Ft (Simpson and Dean, 2002; Parcy, 2005).

Throughout the plant life cycle, bolting and flowering are key life-history traits that exercise far-reaching influence on reproductive suitability, mating patterns and opportunities, gene flow, and evolution (Franks, 2015). Currently, due to global warming, a shift toward early flowering is underway in a wide variety of plant species around the world (Parmesan and Yohe, 2003). Premature bolting and flowering pose a serious problem for production because they decrease both yield and economic value of leafy vegetables; therefore, Ft is a vital trait and a target of selection in crop breeding. A number of studies uncovered the genetic basis of Ft through a quantitative trait locus mapping approach in *Brassica* crops. The *BoFLC2* locus controls Ft in *Brassica oleracea* (Okazaki et al., 2007). The *BrFLC1* and *BrFLC2* genes are associated with variation in Ft in *B. rapa* (Yuan et al., 2009; Zhao et al., 2010). *BnFRI.A3* is associated with Ft in *Brassica napus* (Wang et al., 2011). These studies indicate that homologs of *FLC* and *FRI* play vital roles in the control of Ft in *Brassica* species. In a previous study, we characterized two radish inbred lines, generated by a conventional breeding approach, that exhibit different bolting times under the vernalization conditions. NH-JS1, a late-bolting radish inbred line, is less sensitive to cold treatment than the early-bolting line NH-JS2. Expression of genes related to Ft differs between NH-JS1 and NH-JS2: the repressor *RsFLC1* is expressed at higher levels in NH-JS1, whereas the positive integrator *RsSOC1* is relatively up-regulated in NH-JS2. Although flowering is the most important agricultural trait in cultivation, Ft genes and their associated mechanisms have not been extensively analyzed at a whole-genome scale in radish. Recently, bolting-related miRNAs and their targets were identified in radish using small RNA libraries from leaves at vegetative and reproductive stages (Nie et al., 2015). In addition, differentially expressed genes (DEGs) involved in the transition from vegetative growth to flowering, as well as flowering itself, were identified in radish by RNA-Seq (Nie et al., 2015).

In this study, we performed RNA-Seq and comparative analysis of transcriptomes between two inbred lines with different bolting times, with the goal of identifying regulators of late flowering under vernalization conditions. Our analysis of DEGs related to Ft indicated that most DEGs involved in the vernalization pathway were expressed in patterns that corresponded closely with the different bolting times of the two lines. Negative regulators of the vernalization pathway, such as *RsFLC, RsMAF2, RsSPA1*, and *RsAGL18*, were highly expressed in the late-bolting inbred line NH-JS1, whereas positive regulators of vernalization such as *RsVRN1, RsVIN3*, and *RsAGL19* were relatively highly expressed in the early-bolting line NH-JS2. Based on our findings from RNA-Seq and qPCR analysis, we propose a model regulatory network for the flowering pathway.

## Materials and methods

### Bolting trait analysis of two radish inbred lines

NH-JS1 and NH-JS2, two radish inbred lines developed by NongHyup Seed in Korea (Gyeonggi-do, Anseong, Korea), were used as samples of late-bolting and early-bolting radish plants, respectively. Seeds of each line were sown in sterilized soil and grown under normal growth conditions (23°C, 16 h light/8 h dark) for 2 weeks. For vernalization treatment, 2-week-old plants were grown in the cold room (5 ± 1°C, 12 h light/12 h dark) for 15 or 35 days. After the vernalization periods, the plants were transferred to a normal growth room and grown for 30 days under the same condition. The percentage of bolting plants was measured by counting the bolting plants when the length of the floral axis was ≥1 cm. Ten plants were used for each bolting test, with two biological replicates.

### Plant materials and treatments

To generate samples for RNA-Seq analysis, 2-week-old plants of the NH-JS1 and NH-JS2 inbred lines were exposed to the cold (5 ± 1°C, 12 h light/12 h dark) for 0, 15, or 35 days. Six samples of shoot tissue were collected from each line at the same point in the light/dark cycle. To obtain adequate RNA for each extraction, shoot tissues from three different plants were pooled. Two biological replicates were performed for each vernalization time point. Thus, a total of 12 samples were collected from the two inbred lines. Following harvest, samples were snap-frozen in liquid nitrogen and stored at −80°C until further processing. Total RNA isolation from shoot tissue was performed as described (Jung et al., 2014).

### RNA-Seq library construction and sequencing

Total RNA was prepared for RNA-Seq library construction. mRNA collected from shoot tissues of the two inbred lines was fragmented and used as a template for synthesis of first-strand cDNA using random hexamers and reverse transcriptase. Second-strand cDNA was synthesized using DNA polymerase I (New England BioLabs, Ipswich, MA, USA) and RNase H (Invitrogen, Carlsbad, CA, USA). The resultant cDNA fragments were purified, end-repaired, polyA-tailed, and ligated to index adapters following the Illumina protocol. The ligation products were amplified by PCR and sequenced on an Illumina HiSeq 2000 sequencer system; a 101 bp paired-end sequencing protocol was employed, and two biological replicates were performed for each sample. All raw read data generated in this study were deposited in the GEO of NCBI under accession number GSE89312

### Radish reference-guided assembly and mapping

Raw sequencing data were filtered using standard RNA-Seq parameters (Illumina pipeline). Adapter contamination, low-quality regions, and N-base reads were trimmed from the raw reads, and then reads with a Phred quality score of 31 (Q ≥ 20) or 25 base pairs (bp) were filtered out. These steps were performed using the DynamicTrim and LengthSort programs of the SolexaQA (v.1.13) package (Cox et al., 2010). The cleaned datasets were pooled and mapped to the radish reference gene set. To obtain assembly results, reference-guided assembly was performed using 46,512 genes from the coding sequence regions of the reference genome (Mun et al., 2015). Using the TopHat program, 71,188 assembled unigenes were identified and imposed on the radish reference gene sets. These datasets were pooled and mapped to the radish reference gene set. Mapping was performed using the Bowtie2 (v2.1.0) program (Langmead and Salzberg, 2012) allowing only unique mapping with a maximum of two mismatches; otherwise, the default options were used. The expression levels in each sample were calculated with an in-house script, and read counts for each gene were normalized against library size and rounded to the nearest whole number.

### Functional annotation

Transcripts from RNA-Seq were validated by comparison with gene sequences in the Phytozome database (http://www.phytozome.net/) using BLASTP with *E*-values of at least 1E-10 (BLAST v.2.2.28+) (Altschul et al., 1997). The Blast2GO software v2.8.0 was further used to compare transcripts (≥200 bp) to the non-redundant (NR) databases with thresholds of *E*-value ≤ 1E-05 and 20 BLAST hits (Conesa and Götz, 2008). For Gene Ontology (GO) analysis, the GO database (http://www.geneontology.org/) was downloaded, and the transcripts were annotated to the GO database using BLASTP (*E*-value ≤ 1E-06). GO term annotation was performed using GO classification results from the Map2Slim.pl script. Protein sequences with the highest sequence similarities and cutoffs were retrieved for analysis. Functional enrichment analysis was carried out using DAVID (http://david.abcc.ncifcrf.gov/) and AgriGO (FDR ≤ 0.05) (Du et al., 2010). The transcript lists were further annotated using the TAIR database, and filtered according to default criteria (counts ≥ 2 and EASE score ≤ 0.1). In addition, KEGG pathways were assigned to the sequences by the single-directional best hit method using the KEGG Automatic Annotation Server (Moriya et al., 2007) and DAVID (counts ≥ 10, FDR ≤ 0.05).

### DEGs analysis

Gene expression data were generated from 12 samples from each of two inbred lines. To identify genes differentially expressed during vernalization treatments of each line, raw counts were normalized and analyzed using the DESeq library in R (v3.0) (Anders and Huber, 2010). For a gene to be considered as a DEG, we required |log_2_ (fold change)| ≥ 1. In addition, DEGs were filtered by requiring the adjusted *p*-value (FDR) to be ≤ 0.01. Two types of comparisons were performed: (1) DEGs between the two lines at each time point of vernalization (0, 15, and 35 days); and (2) expression changes of transcripts between vernalization time points: 15 vs. 0 days, and 35 vs. 0 days. Up- and down-regulated transcripts were subjected to a Venn diagram analysis in R.

### Identification of radish homologs of FT

To discover genes related to Ft in our transcriptome, a set of 174 Ft genes was selected as a reference set based on previous literature and studies in *A. thaliana* (Nilsson et al., 1998; Amasino and Michaels, 2010). Published sequences were obtained from the TAIR database (http://www.arabidopsis.org/index.jsp) based on the *Arabidopsis* accession numbers of Ft genes. BLASTn was used to query the 174 Ft genes against the assembled 71,188 genes of radish. Top hits were filtered based on the highest percentage of hit coverage and sequence similarity. Cutoffs were *E*-values ≤ 1E-25 and identity ≥ 65%. Also, the sequences of Ft genes were used to query the protein sequences of radish using BLASTx methods (*E*-value ≤ 1E-20, identity ≥ 50%).

### Validation of a subset of DEGs by qPCR

Expression data obtained by RNA-Seq were validated by quantitative real-time PCR (qPCR) using cDNA synthesized from the same RNA samples used for RNA-Seq analysis. Total RNA was treated with DNase I (Thermo Fisher Scientific, Waltham, MA, USA) to remove genomic DNA. cDNA was synthesized using reverse transcriptase (RevertAid First-strand cDNA Synthesis Kit; Fermentas, Burlington, Canada). qPCR was performed on a CFX Connect™ Real-Time PCR Detection System (Bio-Rad, Hercules, CA, USA) using SYBR Premix Ex Taq (TaKaRa, Tokyo, Japan). DEGs and Ft genes were determined by qPCR using gene-specific primers (Table S3) and were normalized against the corresponding level of *RsACT1*. Three technical repeats were performed for each experiment.

## Results

### Phenotypic variation in bolting time between two radish inbred lines

**Figure 1:**
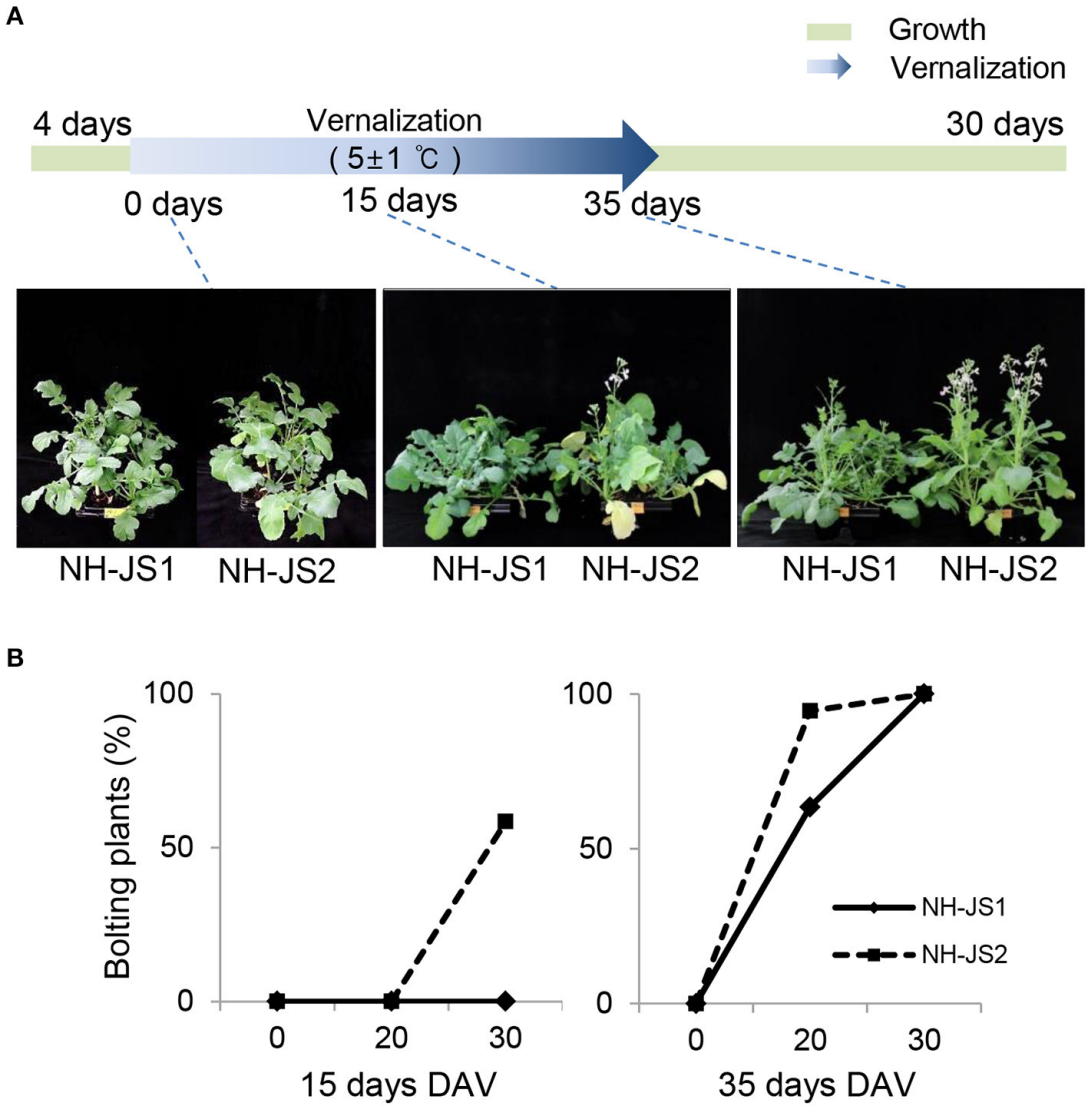
**Phenotypes of NH-JS1 and NH-JS2 inbred lines after vernalization. (A)** Bolting phenotypes of NH-JS1 and NH-JS2 following a vernalization period (0, 15, and 35 days). Seedlings of two inbred lines were germinated in a 23°C growth room for 4 days. The 0 day vernalization time point indicates that the two inbred lines were grown in the 23°C growth room for 30 days without vernalization. For the vernalization treatment, germinated seedlings were grown in a cold room (5 ± 1°C, 12 h light/12 h dark) for 15 or 35 days, and then transferred to a 23°C growth room for 30 days. **(B)** Percentage of bolting plants after vernalization (*n* = 20 plants). DAV, days after vernalization.

First, we recorded the bolting time of the two inbred lines with or without vernalization. After vernalization treatments (0, 15, or 35 days), plants were grown under standard conditions (16 h light/8 h dark, 23°C) for 30 days in soil, and then we examined variations in flowering phenotypes between the two lines. In the absence of vernalization, the two lines were phenotypically similar, i.e., they did not bolt at all. By contrast, 30 days after vernalization treatment, the two lines exhibited dramatic differences, with NH-JS1 exhibiting a relatively late-bolting phenotype (Figure 1A). To further investigate the bolting of the two lines under different conditions, we compared the behavior of the two lines following vernalization treatments of various lengths. When grown for 30 days after 15 days of vernalization treatment, 60% of NH-JS2 bolted, whereas no NH-JS1 plants did. Following 35 days of vernalization conditions, all plants of both lines bolted after 30 days of normal growth. At 20 days of growth after 35 days of vernalization, only 63.5% of NH-JS1 plants bolted, whereas almost all (94.5%) of the NH-JS2 plants did. These results confirmed that NH-JS2 bolts earlier than NH-JS1 in response to vernalization (Figure 1B).

A phenotype is an observable trait caused by differential expression of genes and/or environmental factors. In both inbred lines, vernalization had the most significant effect on the bolting phenotype during growth. Therefore, we predicted that the expression patterns of Ft genes, which we assumed would be closely related to bolting, would differ between the two lines.

### RNA-Seq analysis

To identify transcriptome changes between the two radish inbred lines, we isolated total RNAs from shoot tissues, subjected them to cDNA library construction, and sequenced the libraries on an Illumina HiSeq 2000 Sequencing System (Figure S1). In total, 587,119,316 paired-end reads (101 bp in length) were generated from 12 libraries generated from the two inbred lines. Raw reads were subjected to quality control, and adapter sequences and low-quality reads were trimmed out. Over 79% of clean reads satisfied the following criteria: quality score Q < 20; minimum read length ≥ 25 bp. In total, 237,331,729 and 228,587,020 clean reads were obtained from NH-JS1 and NH-JS2, respectively (Table S1); the average length of clean reads was 87.85 bp. To verify the similarity between two replicate data, normalized counts were used to created a plot of pairs repeat samples (Figure S2). Most samples were highly reproducible between pairs. To assess the proportion of unigenes among the 12 transcriptomes, all clean reads were mapped to the reference set of 71,188 unigenes: 94.35% (241,530,265 reads) reads of NH-JS1 and 94.04% (311,512,975 reads) reads of NH-JS2 mapped to the reference unigenes (Table S2). Among all mapped reads, 284,096,662 (48.45%) reads mapped exactly to the reference unigenes, whereas 268,946,578 (45.75%) reads could map to multiple locations in the reference; only 5% of all reads were unmapped. Overall, these results indicated that all of the transcriptome sets had a high proportion of unigenes and were therefore suitable for further DEG and expression profiling analysis.

### DEGs between two inbred lines during vernalization

**Figure 2:**
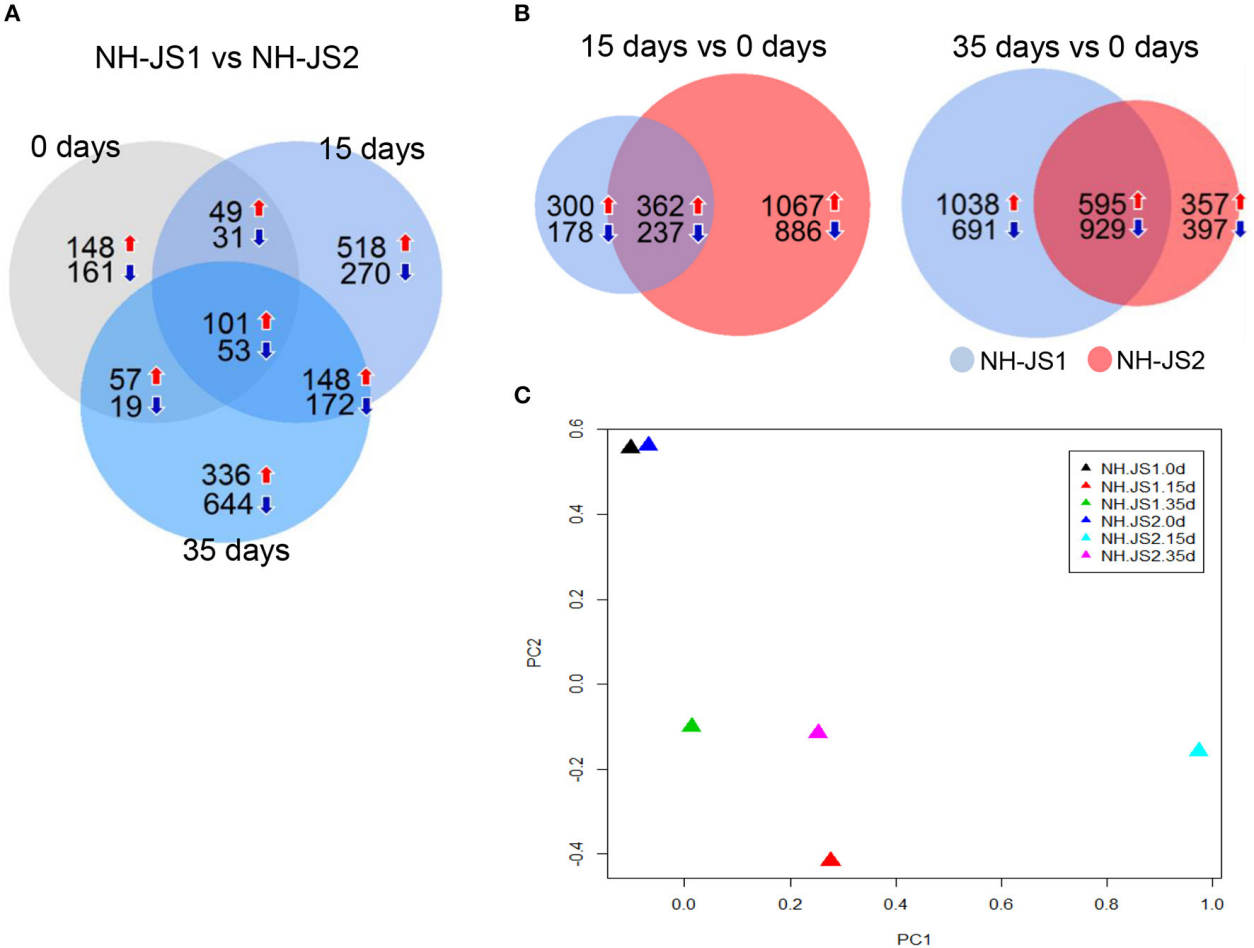
**Comparison of genes differentially expressed between two inbred lines under vernalization conditions. (A)** Differentially expressed genes (DEGs) between the two inbred lines at each vernalization time (0, 15, and 35 days). **(B)** Comparisons of DEGs between 15 and 0 days, and between 35 and 0 days between two inbred lines. Red and blue arrows indicate up-regulation and down-regulation, respectively. Numbers indicate genes differentially expressed in NH-JS1 relative to NH-JS2. **(C)** Principal component analysis of DEGs between two inbred lines under normal and vernalization conditions. Triangles of each color correspond to a different time point. The largest variation between NH-JS1 and NH-JS2 was observed at 15 days of vernalization.

The total set of expressed genes was subjected to DEG analysis using the DESeq package in R to identify individual characteristics of the transcriptomes of the inbred lines. DEGs were selected using the following criteria: FDR ≤ 0.01 and |log_2_(fold change)|≥1. In total, 3491 DEGs were identified between the two inbred lines at each vernalization time point (0, 15, and 35 days). Of these, 154 genes were shared among the three time points, whereas 309, 788, and 980 genes were specific to samples subjected to 0, 15, and 35 days of vernalization, respectively (Figure 2A). Moreover, in both inbred lines, we investigated genes differentially regulated between two time points (vs. 0 days). Between 15 days and 0 days, 1077 genes (662 up-regulated, 415 down-regulated) were differentially expressed in NH-JS1, and 2552 genes (1429 up-regulated, 1123 down-regulated) were differentially expressed in NH-JS2. Between 35 and 0 days, 3253 (1633 up-regulated, 1620 down-regulated) and 2278 (952 up-regulated, 1326 down-regulated) genes were identified as DEGs in NH-JS1 and NH-JS2, respectively (Figure 2B). In the comparison of DEGs between day 15 and day 0, 2.4-fold more DEGs were identified in NH-JS2 than NH-JS1. By contrast, in the comparison between 35 and 0 days, 1.4-fold more DEGs were identified in NH-JS1.

To identify the sets of DEGs that are most relevant to differences in the lines' responses to vernalization, we performed principal component analysis (PCA). The 0 day datasets were similar between the two lines, whereas the 15 and 35 day datasets were dissimilar. The 15 day set from NH-JS2 was the most different from the others. For each inbred line, the results of PCA analysis were consistent with the number of DEGs between the two time points following vernalization treatment, relative to the non-vernalized sample. This suggests that, in both lines, different vernalization times had significantly different effects on the transcription of a subset of transcripts. Thus, these two inbred lines clearly exhibited different expression patterns in response to vernalization treatments of various lengths, as reported in previous studies (Greenup et al., 2011; Villacorta-Martin et al., 2015).

To further investigate the biological pathways active in the two inbred lines, we assigned DEGs from the 15 vs. 0 days comparisons to pathways in the KEGG database. We identified no significantly enriched pathways in NH-JS1, whereas, in NH-JS2, 94 genes were assigned to three pathways: “*glycolysis/gluconeogenesis*” (45 transcripts), “*pyruvate metabolism*” (30 transcripts), and “*nitrogen metabolism*” (19 transcripts). Next, the DEGs from 35 vs. 0 days comparisons were also mapped to the KEGG database. The 352 DEGs from NH-JS1 and the 168 genes from NH-JS2 mapped to 11 and 6 pathways, respectively (Table 1). Five pathways were shared between the two inbred lines, whereas six and one were specific to NH-JS1 and NH-JS2, respectively. The six specific pathways in the NH-JS1 were “*arginine and proline metabolism*,” “*tryptophan metabolism*,” “*glutathione metabolism*,” “*pentose phosphate pathway*,” “*photosynthesis2*,” and “*valine, leucine, and isoleucine biosynthesis*.” The specific pathway in NH-JS2, “*circadian rhythm*,” included 29 transcripts. Further investigation of the DEGs in the two inbred lines may provide clues about phenotypic variation in response to vernalization.

**Table 1:** **Significantly enriched KEGG pathways among differentially expressed genes**.

**Comparison**	**Inbred line**	**KEGG ID**	**Pathway**	**Number of radish genes**
15 vs. 0 days	NH-JS1			
		NA	−	–
	NH-JS2			
		ath00010	Glycolysis/Gluconeogenesis	45
		ath00620	Pyruvate metabolism	30
		ath00910	Nitrogen metabolism	19
35 vs. 0 days	NH-JS1			
		ath00030	Pentose phosphate pathway	23
		ath00195	Photosynthesis1	61
		ath00196	Photosynthesis2	30
		ath00290	Valine, leucine, and isoleucine biosynthesis	31
		ath00330	Arginine and proline metabolism	27
		ath00380	Tryptophan metabolism	17
		ath00480	Glutathione metabolism	28
		ath00620	Pyruvate metabolism	30
		ath00710	Carbon fixation in photosynthetic organisms	46
		ath00910	Nitrogen metabolism	34
		ath00970	Aminoacyl-tRNA biosynthesis	25
	NH-JS2			
		ath00195	Photosynthesis	30
		ath00620	Pyruvate metabolism	23
		ath00710	Carbon fixation in photosynthetic organisms	37
		ath00910	Nitrogen metabolism	30
		ath00970	Aminoacyl-tRNA biosynthesis	19
		ath04712	Circadian rhythm	29

### Identification and expression profiling of Ft genes

To discover radish Ft genes in our transcriptome datasets, we examined Ft genes identified in previous flowering studies in *Arabidopsis*. This analysis revealed 174 Ft genes in *Arabidopsis*, which were confirmed to be involved in flowering pathways. Of 218 putative Ft genes with known homologs in *Arabidopsis*, BLAST search identified 109 Ft genes in our transcriptomes (Table 2). The radish Ft genes were classified into five flowering pathways: “A” (autonomous), “V” (vernalization), “C/L/P” (circadian clock, light signaling, and photoperiod), “D/M” (development and meristem response), and “G/M” (gibberellin signaling and metabolism) (Weigel et al., 1992). The percentages and numbers of genes in each of these pathways were as follows: “C/L/P” (50%, 109 transcripts), “V” (26.1%, 57 transcripts), “A” (10.5%, 23 transcripts), “G/M” (5.9%, 13 transcripts), and “D/M” (5.5%, 12 transcripts).

**Table 2:** **Radish homologs of flowering-time genes**.

**Gene**	**TAIR ID**	**Transcript ID**	**Identity (%)**	***E*-value**	**Pathway**
*AGL18*	AT3G57390	TBIU005421	85.99	0	C/L/P
*AGL18*	AT3G57390	TBIU006406	85.19	0	C/L/P
*AGL19*	AT4G22950	TBIU033103	86.51	0	V
*AGL19*	AT4G22950	TBIU033101	86.51	0	V
*AGL19*	AT4G22950	TBIU040817	88.89	0	V
*AGL24*	AT4G24540	TBIU059222	89.97	0	C/L/P
*AGL24*	AT4G24540	TBIU058842	89.22	0	C/L/P
*AGL24*	AT4G24540	TBIU058841	88.92	0	C/L/P
*AGL24*	AT4G24540	TBIU058840	88.92	0	C/L/P
*AGL24*	AT4G24540	TBIU058839	88.92	0	C/L/P
*AGL31/MAF2*	AT5G65050	TBIU038028	78.66	2E-89	V
*AGL31/MAF2*	AT5G65050	TBIU060196	76.66	3E-88	V
*AGL31/MAF2*	AT5G65050	TBIU041410	87.29	0	V
*AGL31/MAF2*	AT5G65050	TBIU041409	88.84	3E-157	V
*AGL31/MAF2*	AT5G65050	TBIU041406	88.84	3E-157	V
*AP1*	AT1G69120	TBIU053386	85.98	2E-60	D/M
*ATGRP7*	AT2G21660	TBIU039511	82.44	1.00E-121	A
*ATGRP7*	AT2G21660	TBIU008146	79.38	1.00E-62	A
*ATGRP7*	AT2G21660	TBIU008145	79.48	1.00E-63	A
*ATGRP7*	AT2G21660	TBIU008147	79.18	1.00E-60	A
*ATGRP7*	AT2G21660	TBIU034523	83.02	1.00E-61	A
*ATH1*	AT4G32980	TBIU000894	88.29	0	D/M
*ATX1*	AT2G31650	TBIU017557	82.47	0	D/M
*ATX1*	AT2G31650	TBIU017558	81.03	0	D/M
*BRI1*	AT4G39400	TBIU034726	86	0	A
*BRI1*	AT4G39400	TBIU013440	82.08	0	A
*CCA1*	AT2G46830	TBIU056428	86.79	2E-134	C/L/P
*CDF1*	AT5G62430	TBIU001508	81.82	4E-39	C/L/P
*CDF2*	AT5G39660	TBIU055313	81.82	4E-39	C/L/P
*CDF3*	AT3G47500	TBIU048719	80.98	0	C/L/P
*CDF5*	AT1G69570	TBIU032499	81.88	0	C/L/P
*CDF5*	AT1G69570	TBIU018125	79.29	2E-27	C/L/P
*CDF5*	AT1G69570	TBIU018123	79.29	2E-27	C/L/P
*CHE*	AT5G08330	TBIU044938	83	3E-178	C/L/P
*CIB1*	AT4G34530	TBIU050787	78.95	0	C/L/P
*CIR1*	AT5G37260	TBIU002370	82.38	0	C/L/P
*CIR1*	AT5G37260	TBIU002369	82.72	0	C/L/P
*CKB3*	AT3G60250	TBIU008933	89.12	0	C/L/P
*CKB3*	AT3G60250	TBIU008934	89.42	0	C/L/P
*CKB3*	AT3G60250	TBIU014981	85.90	0	C/L/P
*CKB3*	AT3G60250	TBIU063414	88.33	0	C/L/P
*CKB3*	AT3G60250	TBIU047575	89.42	0	C/L/P
*CLF*	AT2G23380	TBIU038214	89.05	0	V
*CLF*	AT2G23380	TBIU038213	87.69	0	V
*CO*	AT5G15840	TBIU047519	82.36	0	C/L/P
*COL5*	AT5G57660	TBIU046921	79.83	0	C/L/P
*COP1*	AT2G32950	TBIU002950	90.38	0	C/L/P
*CRY1*	AT4G08920	TBIU025226	90.27	0	C/L/P
*CRY2*	AT1G04400	TBIU002571	86.53	0	C/L/P
*CRY2*	AT1G04400	TBIU002572	86.69	0	C/L/P
*CRY2*	AT1G04400	TBIU021282	87	0	C/L/P
*CRY2*	AT1G04400	TBIU021281	87	0	C/L/P
*CRY2*	AT1G04400	TBIU021280	87	0	C/L/P
*Cstf64*	AT1G71800	TBIU054341	83.59	0	V
*Cstf64*	AT1G71800	TBIU054340	83.59	0	V
*Cstf77*	AT1G17760	TBIU015459	90	0	V
*CUL4*	AT5G46210	TBIU015701	89.69	0	C/L/P
*CUL4*	AT5G46210	TBIU034296	89.70	0	C/L/P
*EFS*	AT1G77300	TBIU033300	84.35	0	A
*ELF3*	AT2G25930	TBIU017689	83.58	2E-120	C/L/P
*ELF3*	AT2G25930	TBIU017688	76.52	1E-92	C/L/P
*ELF3*	AT2G25930	TBIU017687	76.52	1E-92	C/L/P
*ELF3*	AT2G25930	TBIU060276	79.71	3E-98	C/L/P
*ELF3*	AT2G25930	TBIU060274	81.55	3E-152	C/L/P
*ELF4*	AT2G40080	TBIU064680	94.62	1E-56	C/L/P
*ELF4*	AT2G40080	TBIU049907	91.49	3E-55	C/L/P
*ELF4*	AT2G40080	TBIU050812	92.63	5E-57	C/L/P
*ELF7*	AT1G79730	TBIU005544	83.60	0	C/L/P
*ELF8*	AT2G06210	TBIU051297	91.43	0	C/L/P
*EMF1*	AT5G11530	TBIU055837	77.33	3E-169	V
*EMF2*	AT5G51230	TBIU038305	87.20	0	V
*EMF2*	AT5G51230	TBIU038304	87.20	0	V
*EMF2*	AT5G51230	TBIU038306	87.51	0	V
*ESD1*	AT3G33520	TBIU031116	83.80	0	V
*ESD1*	AT3G33520	TBIU031117	82.59	0	V
*ESD4*	AT4G15880	TBIU053945	88.13	0	V
*ESD4*	AT4G15880	TBIU053944	88.13	0	V
*FCA*	AT4G16280	TBIU061243	87.29	0	A
*FD*	AT4G35900	TBIU030176	80.27	5E-112	C/L/P
*FD*	AT4G35900	TBIU030174	79.09	5E-97	C/L/P
*FD*	AT4G35900	TBIU063145	83.97	0	C/L/P
*FD*	AT4G35900	TBIU063144	82.31	2E-166	C/L/P
*FES*	AT2G33835	TBIU050264	85.32	1E-160	V
*FES*	AT2G33835	TBIU066119	78.68	3E-147	V
*FIO1*	AT2G21070	TBIU055982	82.77	0	C/L/P
*FLC*	AT5G10140	TBIU004737	87.77	0	V
*FLC*	AT5G10140	TBIU055229	86.17	0	V
*FLD*	AT3G10390	TBIU056019	85.46	0	A
*FLK*	AT3G04610	TBIU053081	91.16	0	A
*FLK*	AT3G04610	TBIU053080	91.16	0	A
*FLK*	AT3G04610	TBIU053084	91.16	0	A
*FLK*	AT3G04610	TBIU053083	91.16	0	A
*FLK*	AT3G04610	TBIU053082	87.86	0	A
*FPA*	AT2G43410	TBIU045491	87.28	0	A
*FPA*	AT2G43410	TBIU003165	75.26	0	A
*FPF1*	AT5G24860	TBIU028421	84.55	2E-62	G/M
*FRI*	AT4G00650	NA	NA	NA	V
*FT*	AT1G65480	TBIU006696	87.47	9E-128	I
*FUL*	AT5G60910	TBIU046182	85.98	2E-60	D/M
*FVE*	AT2G19520	TBIU012769	91.17	0	A
*FVE*	AT2G19520	TBIU011465	89.67	0	A
*FVE*	AT2G19520	TBIU003494	92.19	0	A
*FVE*	AT2G19520	TBIU055889	85.12	0	A
*FY*	AT5G13480	TBIU014832	90.82	0	A
*GA2/KS*	AT1G79460	TBIU053644	86.19	0	G/M
*GA2ox2*	AT1G30040	TBIU053462	88.04	0	G/M
*GA2ox2*	AT1G30040	TBIU037959	89.73	0	G/M
*GA2ox6*	AT1G02400	TBIU036436	88.37	0	G/M
*GAI*	AT1G14920	TBIU008587	81.89	0	G/M
*GAI*	AT1G14920	TBIU046318	82.12	0	G/M
*GI*	AT1G22770	TBIU054300	88.30	0	C/L/P
*GID1A*	AT3G05120	TBIU064472	82.55	0	G/M
*GID1A*	AT3G05120	TBIU064471	82.55	0	G/M
*GID1B*	AT3G63010	TBIU043137	86.41	0	G/M
*GID1C*	AT5G27320	TBIU028704	82.45	0	G/M
*HUA2*	AT5G23150	TBIU046200	83.18	0	V
*HUA2*	AT5G23150	TBIU046198	83.18	0	V
*HUA2*	AT5G23150	TBIU033617	85.14	0	V
*HUA2*	AT5G23150	TBIU033616	85.14	0	V
*HUB1*	AT2G44950	TBIU022601	88.70	0	V
*HUB1*	AT2G44950	TBIU045258	88.41	0	V
*HUB1*	AT2G44950	TBIU045256	87.80	0	V
*HUB1*	AT2G44950	TBIU045257	88.19	0	V
*HUB2*	AT1G55250	TBIU005540	89.97	0	V
*JMJ14*	AT4G20400	TBIU005695	86.35	0	C/L/P
*JMJ14*	AT4G20400	TBIU027036	84.13	0	C/L/P
*LD*	AT4G02560	TBIU060988	86.22	0	A
*LFY*	AT5G61850	NA	NA	NA	I
*LHY*	AT1G01060	TBIU002252	81.64	0	C/L/P
*LHY*	AT1G01060	TBIU002251	81.64	0	C/L/P
*LKP2*	AT2G18915	TBIU044770	85.77	0	C/L/P
*LKP2*	AT2G18915	TBIU044765	85.77	0	C/L/P
*LKP2*	AT2G18915	TBIU044766	85.94	0	C/L/P
*LKP2*	AT2G18915	TBIU044769	82.51	0	C/L/P
*LUX/PCL1*	AT3G46640	TBIU006035	80.54	2E-91	C/L/P
*LWD1/LWD2*	AT1G12910	TBIU027456	85.41	0	C/L/P
*MAF3*	AT5G65060	TBIU059941	70.10	3.00E-79	V
*MYB33*	AT5G06100	TBIU055380	75.12	4E-125	G/M
*NFYA1*	AT5G12840	TBIU026781	82.41	0	C/L/P
*NFYA1*	AT5G12840	TBIU063072	85.48	2E-136	C/L/P
*NFYA4*	AT2G34720	TBIU046301	87.35	6E-140	C/L/P
*NFYA4*	AT2G34720	TBIU046298	87.24	6E-140	C/L/P
*NFYA4*	AT2G34720	TBIU053915	77.60	2E-55	C/L/P
*NFYA4*	AT2G34720	TBIU053916	77.60	2E-55	C/L/P
*NFYB2*	AT5G47640	TBIU015167	87.43	9E-168	C/L/P
*NFYB2*	AT5G47640	TBIU015166	87.43	9E-168	C/L/P
*NFYB2*	AT5G47640	TBIU039384	86.76	1E-170	C/L/P
*NFYB2*	AT5G47640	TBIU039382	86.76	1E-170	C/L/P
*NFYB2*	AT5G47640	TBIU039381	86.76	1E-170	C/L/P
*NFYB2*	AT5G47640	TBIU039383	86.76	1E-170	C/L/P
*NFYB3*	AT4G14540	TBIU054661	74.85	2.00E-38	C/L/P
*NFYC9*	AT1G08970	TBIU020621	85.67	0	C/L/P
*PFT1*	AT1G25540	TBIU063731	88.42	0	C/L/P
*PFT1*	AT1G25540	TBIU063730	88.42	0	C/L/P
*PFT1*	AT1G25540	TBIU063732	88.84	0	C/L/P
*PHYA*	AT1G09570	TBIU022457	88.25	0	C/L/P
*PHYA*	AT1G09570	TBIU022456	88.25	0	C/L/P
*PHYB*	AT2G18790	TBIU006411	86.60	0	C/L/P
*PHYB*	AT2G18790	TBIU006412	86.42	0	C/L/P
*PHYB*	AT2G18790	TBIU052711	84.91	0	C/L/P
*PHYC*	AT5G35840	TBIU014324	87.85	0	C/L/P
*PHYC*	AT5G35840	TBIU014323	87.95	0	C/L/P
*PHYE*	AT4G18130	TBIU021641	84.82	0	C/L/P
*PIE1*	AT3G12810	TBIU054149	88.19	0	C/L/P
*PIF3*	AT1G09530	TBIU022387	81.68	0	C/L/P
*PIF3*	AT1G09530	TBIU022386	81.44	0	C/L/P
*PIF3*	AT1G09530	TBIU061019	83.52	8E-63	C/L/P
*PRR1/TOC1*	AT5G61380	TBIU063569	88.53	0	C/L/P
*PRR3*	AT5G60100	TBIU002967	88.45	0	C/L/P
*PRR5*	AT5G24470	TBIU054683	80.38	2E-179	C/L/P
*PRR5*	AT5G24470	TBIU031542	84.99	0	C/L/P
*PRR5*	AT5G24470	TBIU018984	84.99	0	C/L/P
*RFI2*	AT2G47700	TBIU025658	79.42	0	C/L/P
*SAP18*	AT2G45640	TBIU003917	86.06	4E-140	D/M
*SAP18*	AT2G45640	TBIU017539	86.24	9E-142	D/M
*SDG26*	AT1G76710	TBIU028878	86.89	0	V
*SDG26*	AT1G76710	TBIU028877	86.89	0	V
*SLY1*	AT4G24210	TBIU059296	82.91	1E-95	G/M
*SMZ*	AT3G54990	TBIU035344	87.20	0	C/L/P
*SOC1*	AT2G45660	TBIU065173	94.27	0	I
*SOC1*	AT2G45660	TBIU057467	93.95	0	I
*SPA1*	AT2G46340	TBIU023088	87.32	0	C/L/P
*SPA3*	AT3G15354	TBIU006466	84.72	0	C/L/P
*SPA3*	AT3G15354	TBIU006465	83.24	0	C/L/P
*SPA3*	AT3G15354	TBIU024047	84.72	0	C/L/P
*SPL3*	AT2G33810	TBIU008541	83.10	1E-99	D/M
*SPL4*	AT1G53160	TBIU015321	84.27	1E-131	D/M
*SPL4*	AT1G53160	TBIU010825	85.59	7E-28	D/M
*SPL4*	AT1G53160	TBIU053283	80.32	7E-74	D/M
*SPL9*	AT2G42200	TBIU040688	85.51	0	D/M
*SVP*	AT2G22540	TBIU062055	89.04	0	V
*SVP*	AT2G22540	TBIU023068	89.04	0	V
*TEM1*	AT1G25560	TBIU011903	81.84	0	C/L/P
*TEM2*	AT1G68840	TBIU004005	75.05	9E-106	C/L/P
*TEM2*	AT1G68840	TBIU026467	78.52	4.00E-134	C/L/P
*TFL2*	AT5G17690	TBIU003330	81.91	3E-146	C/L/P
*TFL2*	AT5G17690	TBIU003331	81.91	3E-146	C/L/P
*TIC*	AT3G22380	TBIU051595	81.70	0	C/L/P
*TIC*	AT3G22380	TBIU047831	81.77	0	C/L/P
*TOE1*	AT2G28550	TBIU064891	88.86	5E-144	C/L/P
*UBC1*	AT1G14400	TBIU046552	89.98	3E-171	V
*UBC1*	AT1G14400	TBIU041501	94.34	0	V
*UBC1*	AT1G14400	TBIU024449	94.34	0	V
*UBC1*	AT1G14400	TBIU024448	94.34	0	V
*UBC1*	AT1G14400	TBIU063982	94.34	0	V
*VIN3*	AT5G57380	TBIU064307	81.59	0	V
*VIN3*	AT5G57380	TBIU064308	81.58	0	V
*VIN3*	AT5G57380	TBIU064309	82.02	0	V
*VIN3*	AT5G57380	TBIU035197	84.80	0	V
*VIP4*	AT5G61150	TBIU053576	84.94	0	V
*VIP4*	AT5G61150	TBIU046204	83.90	0	V
*VIP5*	AT1G61040	TBIU044708	82.86	0	V
*VRN1*	AT3G18990	TBIU020397	88.96	0	V
*VRN1*	AT3G18990	TBIU003967	86.27	0	V
*VRN2*	AT4G16845	TBIU010681	74.81	0	V
*VRN2*	AT4G16845	TBIU010680	74.81	0	V
*VRN5*	AT3G24440	TBIU055842	80.08	0	V
*WNK1*	AT3G04910	TBIU064959	89.15	0	C/L/P

*- C/L/P: Circadian Clock, Light Signaling, and Photoperiod Pathways*.

*- D/M: Development and Meristem Response*.

*- G/M: Gibberellin Signaling and Metabolism*.

*- I: Floral Integrator*.

*- V: Vernalization Pathway*.

**Figure 3:**
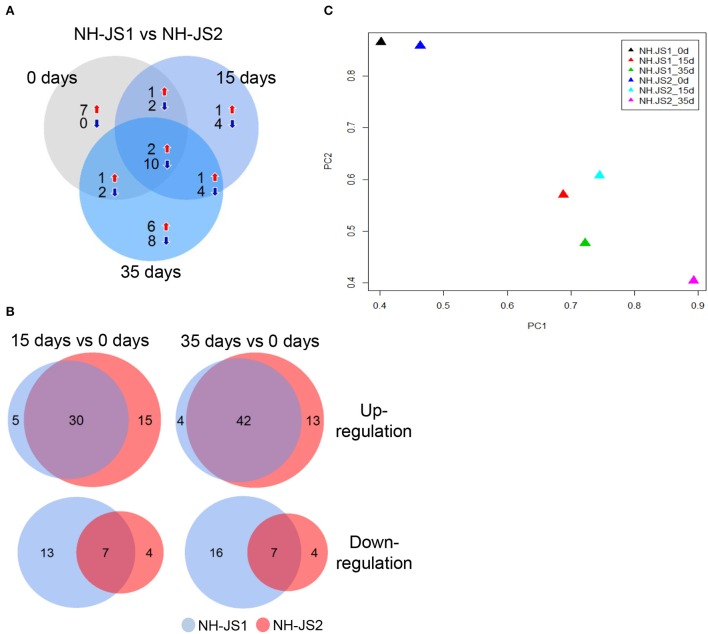
**Comparison of flowering-time genes differentially expressed between two inbred lines under vernalization conditions. (A)** Differentially expressed genes (DEGs) compared between the two inbred lines at each vernalization time (0, 15, and 35 days). **(B)** Comparisons of flowering-time DEGs between 15 and 0 days, and between 35 and 0 days, in the two inbred lines. Red and blue arrows indicate up-regulation and down-regulation, respectively. Numbers indicate genes differentially expressed in NH-JS1 relative to NH-JS2. **(C)** Principle component analysis of flowering-time DEGs between two inbred lines in the normal and vernalization conditions. Each color of triangle corresponds to a different time point in the two inbred lines. The largest variation between NH-JS1 and NH-JS2 was observed at 35 days of vernalization.

To identify DEGs related to the flowering pathway, we identified DEGs between the two inbred lines from among the 218 putative Ft genes. In total, 26 and 58 Ft genes were identified as DEGs at the three vernalization time points in the NH-JS1 and NH-JS2, respectively: 11 and 14 genes at 0 days, 5 and 20 genes at 15 days, and 10 and 24 genes at 35 days (Figure 3A). Interestingly, 12 transcripts were consistently detected as DEGs at all vernalization time points. Furthermore, in both lines, we identified DEGs between vernalization time points; in the comparison between 15 and 0 days, 35 and 45 genes were up-regulated and 20 and 11 were down-regulated in NH-JS1 and NH-JS2, respectively. In the comparison between 35 and 0 days, 46 and 55 transcripts were up-regulated, and 23 and 11 were down-regulated in NH-JS1 and NH-JS2, respectively (Figure 3B). These six sets of Ft-related DEGs were analyzed by PCA. This analysis revealed that the datasets from 0 and 15 days of vernalization were similar between the two inbred lines, whereas the sets from 35 days of vernalization were divergent. The 35 day set from NH-JS2 was the most different from the others (Figure 3C). These results were consistent with the results of DEG analysis of Ft genes between the two lines. Interestingly, most of the up-regulated genes (16 of the 19 genes) in NH-JS1 were repressors of flowering, whereas the down-regulated genes in NH-JS1 consisted of 18 enhancer and 12 repressor genes. *RsMAF2* (TBIU060196) and *RsSPA1* (TBIU023088), which are repressors of the flowering pathway, were always up-regulated, whereas the positive regulators of flowering *RsAGL19* (TBIU033101, TBIU033103, and TBIU040817), *RsNFYA4* (TBIU046301 and TBIU046298), and *RsSOC1* (TBIU065173 and TBIU057467) were consistently down-regulated in NH-JS1, regardless of vernalization time. In addition, most down-regulated genes in NH-JS1 were flowering enhancers (18 of 30 genes), with the exception of *RsELF3* (5 of 30 genes) and *RsELF4* (3 of 30 genes) (Table 3).

**Table 3:** **Radish genes related to flowering time and their expression profile during vernalization**.

**ID**	**TAIR ID**	**Gene**	**Flowering enhancer/repressor**	**Regulation**	**Time point**	**Pathway**	**Identity (%)**	***E*-value**
TBIU060196	AT5G65050	*MAF2/AGL31*	Repressor	Up	0/15/35	V	78.66	2.00E-89
TBIU023088	AT2G46340	*SPA1*	Repressor	Up	0/15/35	C/L/P	87.32	0
TBIU002252	AT1G01060	*LHY*	Enhancer	Up	0	C/L/P	81.64	0
TBIU002251	AT1G01060	*LHY*	Enhancer	Up	0	C/L/P	81.64	0
TBIU038028	AT5G65050	*MAF2/AGL31*	Repressor	Up	0	V	78.66	2.00E-89
TBIU037959	AT1G30040	*GA2ox2*	Repressor	Up	0	G/M	88.04	0
TBIU022387	AT1G09530	*PIF3*	Repressor	Up	0	C/L/P	81.68	0
TBIU022386	AT1G09530	*PIF3*	Repressor	Up	0	C/L/P	81.68	0
TBIU039152	AT5G61380	*PRR1*	Repressor	Up	0	C/L/P	88.53	0
TBIU035344	AT3G54990	*SMZ*	Repressor	Up	15	C/L/P	87.2	0
TBIU005421	AT3G57390	*AGL18*	Repressor	Up	35	C/L/P	85.99	0
TBIU048719	AT3G47500	*CDF3*	Repressor	Up	35	C/L/P	80.98	0
TBIU055229	AT5G10140	*FLC*	Repressor	Up	35	V	87.77	0
TBIU041410	AT5G65050	*MAF2/AGL31*	Repressor	Up	35	V	78.66	2.00E-89
TBIU041409	AT5G65050	*MAF2/AGL31*	Repressor	Up	35	V	78.66	2.00E-89
TBIU041406	AT5G65050	*MAF2/AGL31*	Repressor	Up	35	V	78.66	2.00E-89
TBIU059941	AT5G65060	*MAF3*	Repressor	Up	0/15	V	66.5	3.00E-79
TBIU006406	AT3G57390	*AGL18*	Repressor	Up	0/35	C/L/P	85.99	0
TBIU028421	AT5G24860	*FPF1*	Enhancer	Up	15/35	G/M	84.55	2.00E-62
TBIU065173	AT2G45660	*SOC1*	Enhancer	Down	0/15/35	I	94.27	0
TBIU057467	AT2G45660	*SOC1*	Enhancer	Down	0/15/35	I	94.27	0
TBIU017689	AT2G25930	*ELF3*	Repressor	Down	0/15/35	C/L/P	81.55	3.00E-152
TBIU017688	AT2G25930	*ELF3*	Repressor	Down	0/15/35	C/L/P	81.55	3.00E-152
TBIU017687	AT2G25930	*ELF3*	Repressor	Down	0/15/35	C/L/P	81.55	3.00E-152
TBIU033103	AT4G22950	*AGL19*	Enhancer	Down	0/15/35	V	88.89	0
TBIU033101	AT4G22950	*AGL19*	Enhancer	Down	0/15/35	V	88.89	0
TBIU040817	AT4G22950	*AGL19*	Enhancer	Down	0/15/35	V	88.89	0
TBIU046301	AT2G34720	*NFYA4*	Enhancer	Down	0/15/35	C/L/P	87.24	6.00E-140
TBIU046298	AT2G34720	*NFYA4*	Enhancer	Down	0/15/35	C/L/P	87.24	6.00E-140
TBIU049907	AT2G40080	*ELF4*	Repressor	Down	15	C/L/P	91.49	3.00E-55
TBIU030174	AT4G35900	*FD*	Enhancer	Down	15	C/L/P	83.97	0
TBIU062055	AT2G22540	*SVP*	Repressor	Down	15	V	89.04	0
TBIU011903	AT1G25560	*TEM1*	Repressor	Down	15	C/L/P	81.84	0
TBIU047519	AT5G15840	*CO*	Enhancer	Down	35	C/L/P	82.36	0
TBIU060276	AT2G25930	*ELF3*	Repressor	Down	35	C/L/P	81.55	3.00E-152
TBIU060274	AT2G25930	*ELF3*	Repressor	Down	35	C/L/P	81.55	3.00E-152
TBIU035197	AT5G57380	*VIN3*	Enhancer	Down	35	V	81.59	0
TBIU063145	AT4G35900	*FD*	Enhancer	Down	35	D/M	83.97	0
TBIU063144	AT4G35900	*FD*	Enhancer	Down	35	D/M	83.97	0
TBIU046318	AT1G14920	*GAI*	Enhancer	Down	35	G/M	82.12	0
TBIU061019	AT1G09530	*PIF3*	Repressor	Down	35	C/L/P	81.68	0
TBIU053386	AT1G69120	*AP1*	Enhancer	Down	0/15	D/M	85.98	2.00E-60
TBIU046182	AT5G60910	*FUL*	Enhancer	Down	0/15	D/M	85.98	2.00E-60
TBIU010825	AT1G53160	*SPL4*	Enhancer	Down	0/35	D/M	84.27	1.00E-131
TBIU053283	AT1G53160	*SPL4*	Enhancer	Down	0/35	D/M	84.27	1.00E-131
TBIU064680	AT2G40080	*ELF4*	Repressor	Down	15/35	C/L/P	94.62	1.00E-56
TBIU050812	AT2G40080	*ELF4*	Repressor	Down	15/35	C/L/P	92.63	5.00E-57
TBIU026467	AT1G68840	*TEM2*	Repressor	Down	15/35	C/L/P	85.03	3.00E-155
TBIU006035	AT3G46640	*LUX/PCL1*	Enhancer	Down	15/35	C/L/P	80.54	2.00E-91

*- C/L/P: Circadian Clock, Light Signaling, and Photoperiod Pathways*.

*- D/M: Development and Meristem Response*.

*- G/M: Gibberellin Signaling and Metabolism*.

*- I: Floral Integrator*.

*- V: Vernalization Pathway*.

To identify Ft genes that were differentially regulated between each time point among the Ft DEGs, we investigated the expression profiles of the Ft DEGs based on RNA-Seq data. Fourteen Ft genes were identified based on sequential expression changes between the lines during vernalization (Table 4). *RsNFYA4, RsSOC1*, and *RsSPA1* exhibited > 2-fold expression differences between the two lines at all-time points. *RsFUL, RsMAF3, RsPIF3*, and *RsSPL4* exhibited expression differences between the lines at 0 days: *RsELF4, RsLUX/PCL1*, and *RsSVP* were differentially expressed at 15 days; and *RsCDF3, RsELF3, RsMAF2/AGL31*, and *RsVIN3* genes were differentially expressed at 35 days. The resultant changes in Ft gene transcription are likely to be involved in differences in bolting in response to vernalization.

**Table 4:** **Differential expression of flowering time–related genes during cold exposure (0, 15, and 35 days)**.

**Gene**	**TAIR ID**	**Homolog**	**Protein identity (%)**	**Expression profile**		**NH-JS1**			**NH-JS2**			**Comparison periods**
				**NH-JS1**	**NH-JS2**	**0 days**	**15 days**	**35 days**	**0 days**	**15 days**	**35 days**	
*NFYA4*	AT2G34720	TBIU046301	83	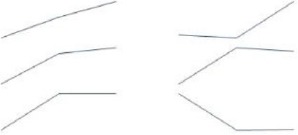		8.55	9.14	9.62	10.45	10.39	11.11	All
*SOC1*	AT2G45660	TBIU065173	95			7.03	9.87	10.46	10.19	12.40	12.16	**
*SPA1*	AT2G46340	TBIU023088	82			11.72	12.08	12.08	10.53	9.76	9.78	
*FUL*	AT5G60910	TBIU046182	93	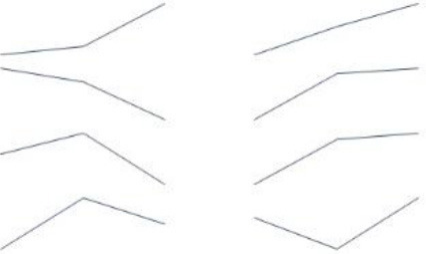		3.91	4.55	7.98	6.39	7.11	7.73	0 days
*MAF3*	AT5G65060	TBIU059941	67			7.60	7.44	7.01	5.66	7.94	8.22	**
*PIF3*	AT1G09530	TBIU022387	76			8.36	8.73	7.85	7.26	8.33	8.48	
*SPL4*	AT1G53160	TBIU010825	71			5.32	5.89	5.60	6.69	6.32	6.93	
*ELF4*	AT2G40080	TBIU049907	91	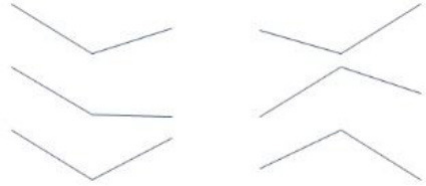		8.76	7.74	8.26	9.02	8.91	9.14	15 days
*LUX/PCL1*	AT3G46640	TBIU006035	77			7.91	6.62	6.56	7.46	8.18	7.80	
*SVP*	AT2G22540	TBIU062055	90			9.86	9.45	9.79	10.45	10.69	10.39	
*CDF3*	AT3G47500	TBIU024827	77	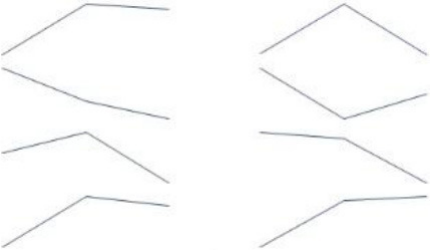		10.89	11.76	11.66	10.56	11.61	10.54	35 days
*ELF3*	AT2G25930	TBIU060276	59			9.55	8.68	8.21	9.86	8.76	9.29	
*MAF2/AGL31*	AT5G65050	TBIU041410	63			9.68	9.87	9.41	9.71	9.45	7.79	
*VIN3*	AT5G57380	TBIU035197	77			4.93	8.22	7.60	5.32	8.30	8.63	

### Validation of DEGs and expression profiling of Ft DEGs by qPCR

**Figure 4:**
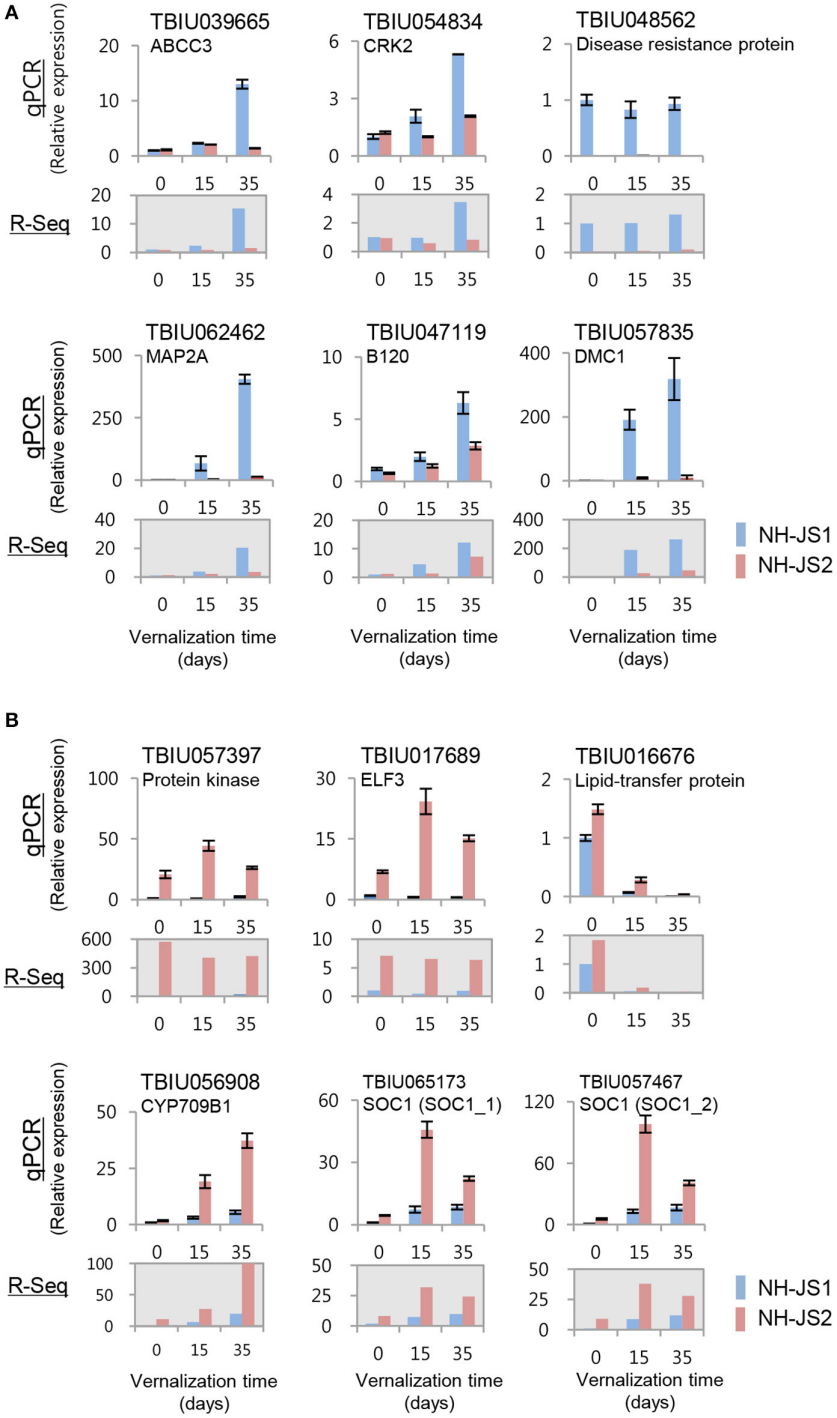
**Validation of RNA-Seq data by qPCR**. cDNA synthesized from total RNA extracted from shoots of NH-JS1 and NH-JS2 inbred lines, following vernalization for 0, 15, or 35 days. **(A)** Up-regulated and **(B)** down-regulated genes in NH-JS1 vs. NH-JS2 were selected from RNA-Seq data for validation. qPCR values were normalized against the corresponding level of *RsACT1* (actin). For each gene in both the qPCR and RNA-Seq analysis, the expression level from NH-JS1 on day 0 was defined as “1.”

To evaluate the DEGs identified by RNA-Seq analysis, we performed qPCR. For this purpose, we selected 12 genes that exhibited the highest difference in expression levels between NH-JS1 and NH-JS2. Six of these transcripts (TBIU039665, TBIU054834, TBIU048562, TBIU062462, TBIU047119, and TBIU057835) were up-regulated, and the other six (TBIU057397, TBIU017689, TBIU016676, TBIU056908, TBIU065173, and TBIU057467) were down-regulated, in NH-JS1 vs. NH-JS2. With the exceptions of TBIU048562 and TBIU016676, all of these genes were strongly influenced by vernalization treatments. The qPCR analysis yielded expression patterns consistent with the RNA-Seq data. In particular, the transcript levels of *RsELF3* (TBIU017689) and *RsSOC1* (TBIU065173, TBIU057467), which are intimately involved in flowering, were, respectively, 43- and 8-fold more highly expressed in NH-JS2 than in NH-JS1 (Figure 4).

**Figure 5:**
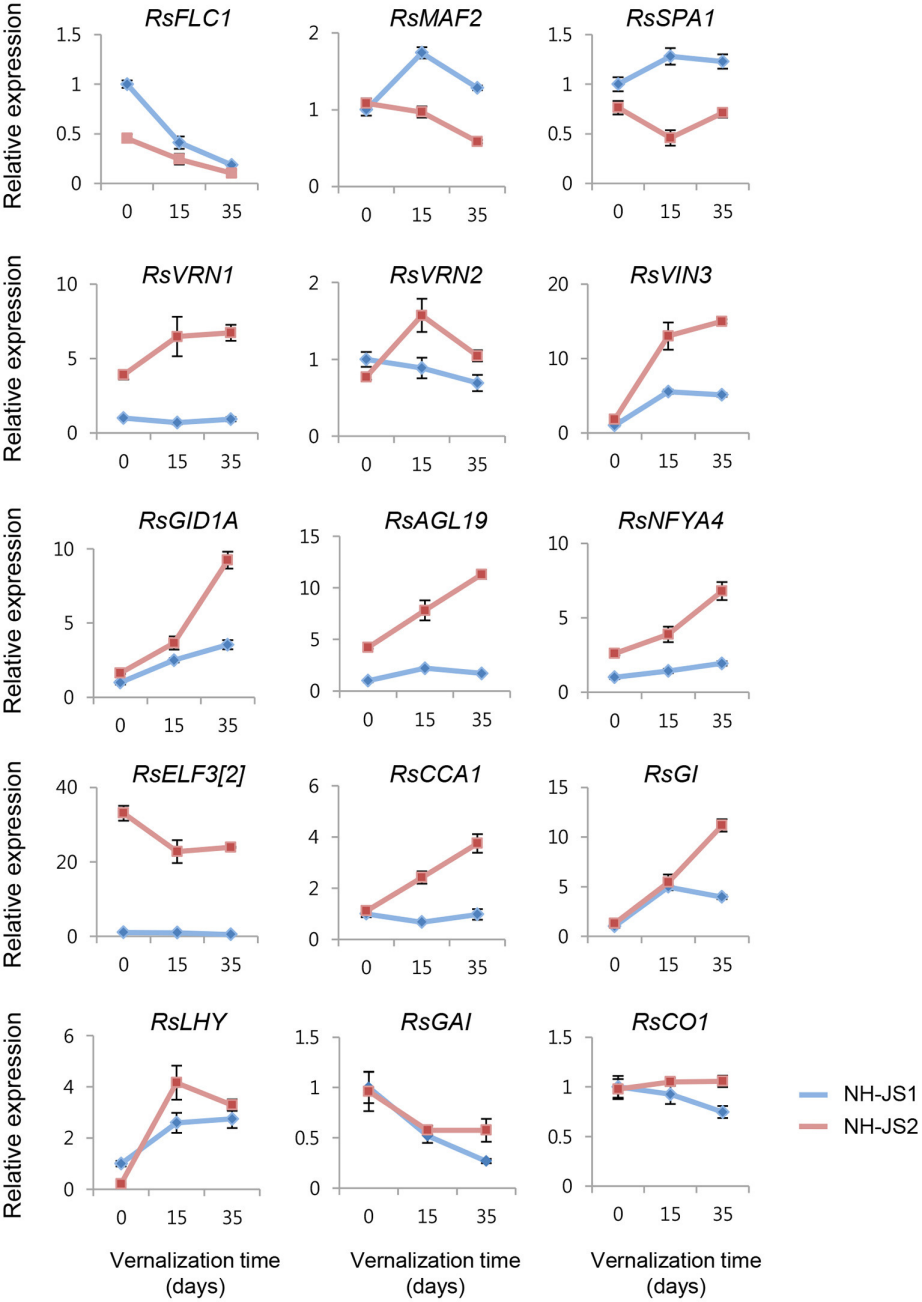
**Differential expression between the two inbred lines of major flowering-time genes during vernalization**. cDNA synthesized from total RNA extracted from the shoots of NH-JS1 and NH-JS2 inbred lines after vernalization times of 0, 15, or 35 days. qPCR expression level was normalized against the corresponding level of *RsACT1*. For each, the expression level from NH-JS1 on day 0 was defined as “1.”

In addition, we performed qPCR analysis to investigate the expression levels of major Ft-regulating genes, which play essential roles in various flowering pathways. A major determinant of Ft, *RsFLC1* (FLOWER LOCUS C; suppressor of flowering), was reduced during vernalization in both lines. Before vernalization treatment, *RsFLC1* was 2-fold more highly expressed in NH-JS1; however, the difference in expression level was gradually reduced after 15 and 35 days of vernalization. As in the Ft DEG analysis, most DEGs involved in the vernalization pathway matched with the different bolting phenotype between the lines. Therefore, we also performed qPCR to investigate the expression levels of major Ft genes involved in the vernalization pathway. Interestingly, *RsVRN1* and *RsVRN2*, which are positive regulators of flowering, exhibited different expression levels and opposing expression patterns between the NH-JS1 and NH-JS2 lines. In NH-JS2, the expression levels of these genes increased after vernalization, whereas in NH-JS1 they remained constant or decreased. VRN1, an essential regulator of the floral transition, is gradually up-regulated by vernalization and negatively regulates VRN2 expression (Yan et al., 2003). In particular, *RsVRN1* was 3 to 7-fold more highly expressed in NH-JS2 than NH-JS1 during vernalization, but its expression was not induced in NH-JS1 in response to cold exposure or in the absence of vernalization (Figure 5). Expression levels of *RsVIN3*, a flowering enhancer, as well as the GA pathway-regulated genes *RsGID1A, RsAGL19*, and *RsNFYA4*, were highly induced by vernalization treatment in both lines. Moreover, differences in the expression levels between the two lines were increased up to 3-fold during vernalization treatment in NH-JS2. In addition, we checked the expression levels of photoperiod pathway genes (*RsELF3, RsCCA1, RsGI, RsLHY*, and *RsFPA*). These genes were expressed in patterns similar to those previously described, and most were more highly expressed in NH-JS2 than NH-JS1. Expression of *RsFPA* and *RsLHY* was induced similarly by vernalization treatment in both lines (Figure 5). To address the role of the major FT-regulating genes, *RsFLC* and *RsSOC1*, in the GA-regulated flowering pathway, the gene expression levels were analyzed following exogenous GA treatment in two inbred lines under or not under vernalization conditions. The expression levels of the genes differed very little between the two lines in the early GA response, whereas they significantly differed between the two lines when the bolting phenotype was exhibited. NH-JS2 reacted more sensitively to GA treatment than late-bolting NH-JS1 at 20 days after GA application (data not shown). High-throughput RNA-Seq analysis is now underway to investigate the difference in response to GA treatment between the two lines.

### The transcriptional regulatory network underlying differences in bolting time between the two lines

**Figure 6:**
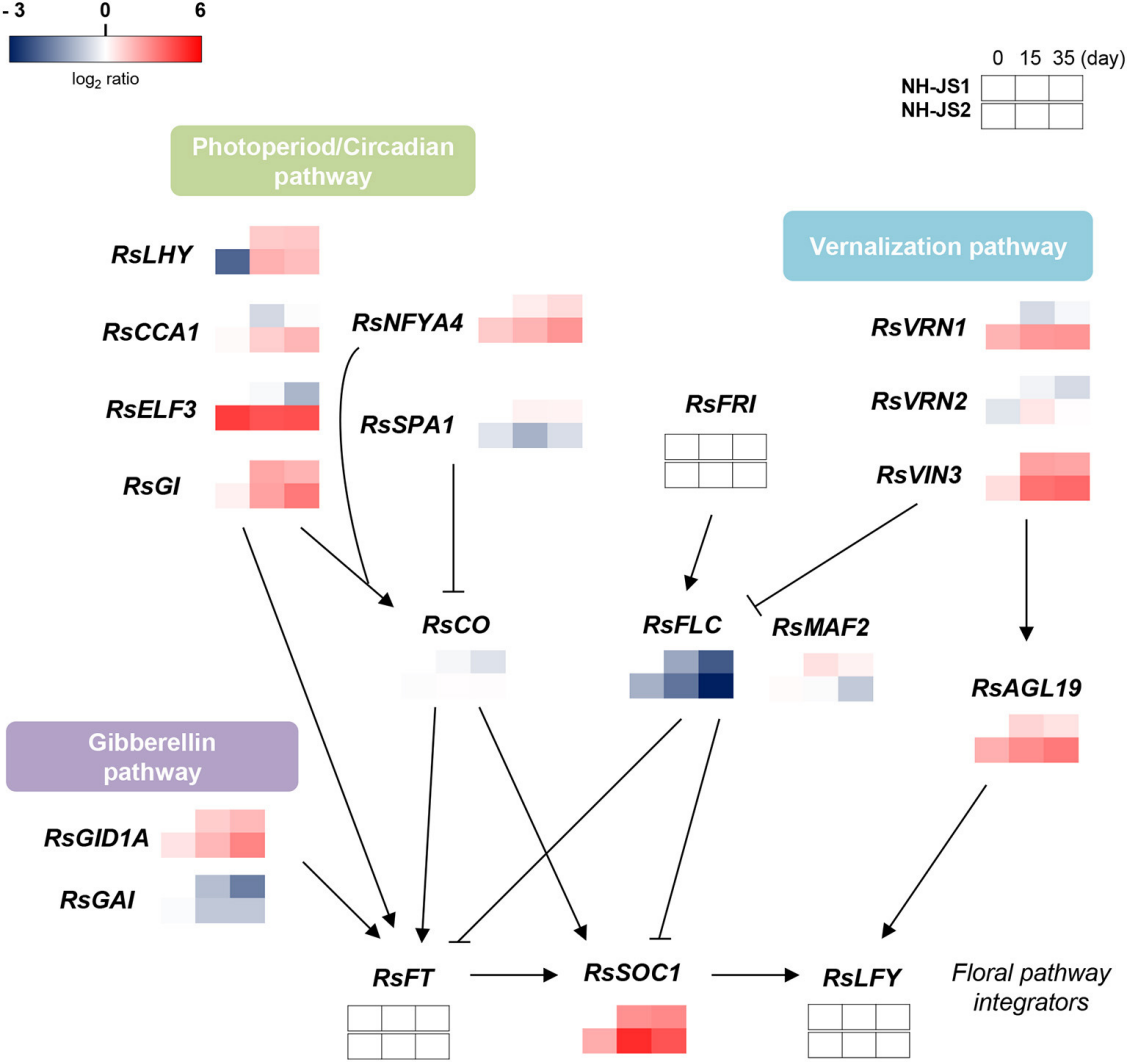
**Gene regulatory network controlling flowering time in radish**. The schema represents the regulatory network of Ft-related genes in late-bolting NH-JS1, in comparison with NH-JS2, under the differential vernalization conditions. The model is based on data obtained by qPCR. Gene expression levels were normalized to the expression levels in non-vernalized NH-JS1 plants. Red indicates higher expression and blue indicates lower expression relative to NH-JS1 on day 0. Arrows indicate transcriptional activation, whereas bars indicate transcriptional repression.

To elucidate the overall flowering pathway governing differences in bolting time, we propose a model regulatory network based on differences in Ft gene expression (as determined by qPCR) between the two inbred lines. We divided the Ft DEGs into three major flowering pathways: vernalization, photoperiod/circadian, and gibberellin. *RsFLC* was negatively regulated under vernalization and was expressed at higher levels in the late-bolting NH-JS1, whereas another repressor, *RsMAF2*, was positively regulated in response to vernalization and was also highly expressed in the NH-JS1 line. On the other hand, the enhancers of the vernalization pathway, *RsVRN1, RsVIN3*, and *RsAGL19*, were positively regulated upon vernalization and expressed more highly in the early-bolting NH-JS2 line. *RsVRN2*, a floral repressor, did not exhibit significant changes in expression. Most DEGs involved in the photoperiod pathway (*RsCCA1, RsLHY, RsELF3*, and *RsGI*) were remarkably highly expressed in early-bolting NH-JS2; however, *RsCO* was not highly expressed. *RsNFYA4*, an enhancer of the photoperiod pathway, also exhibited elevated expression in the early-bolting line. The DELLA domain protein *RsGAI*, a negative regulator of flowering, exhibited reduced expression in NH-JS1, and a repressor of DELLA proteins, *RsGID1A*, was also down-regulated in this line (Figure 6).

## Discussion

In this study, we investigated the flowering traits of two radish inbred lines under vernalization conditions. Line NH-JS1 was late-bolting following vernalization treatment, whereas NH-JS2 was early-bolting. Our results confirmed that vernalization was necessary for radish bolting and that the vernalization periods required to induce flowering differed between the two inbred lines (Figure 1). RNA-Seq was conducted to identify important genes involved in late bolting and elucidate the molecular network that regulates the flowering pathway in this crop plant. Using radish shoot tissue from young plants, we analyzed individual transcriptomes during vernalization by high-throughput RNA sequencing. Comparative analysis among the radish transcriptomes revealed significant characteristics that were biologically reproducible (Figure S2). From 40 to 71 million reads were generated per samples (100 bp, paired-end), representing 11 × coverage of the radish genome. Previous studies reported approximately 70,000 unigenes in radish (Wang et al., 2013a,b; Wu et al., 2015). Consistent with this, our transcriptome analysis identified 71,188 genes despite using only shoot tissue for the RNA-Seq analysis.

### Differential expression of Ft-related genes governs bolting in response to vernalization

Genome-wide DEG analysis revealed that vernalization treatments of 15 and 35 days had significantly different effects on the transcription of a subset of genes between the two inbred lines (Figure 2), and KEGG pathway analysis revealed similar trends (Table 1). The pathway analysis showed that gene expression related to nitrogen and pyruvate metabolism is significantly up-regulated during vernalization in radish. Further investigation of DEGs in the two inbred lines may provide clues about phenotypic variations related to vernalization.

By comparison with known Ft-related genes in *Arabidopsis*, we identified 218 radish Ft genes in our transcriptome analysis (Nilsson et al., 1998; Table 2), of which 49 were differentially expressed between NH-JS1 and NH-JS2. Thus, about 80% of Ft genes were expressed at similar levels in both lines, suggesting that the 49 Ft-related DEGs provide several hints regarding the molecular basis of late-bolting time in NH-JS1. First, expression of repressors of flowering such as *RsFLC*1 was elevated in NH-JS1, and 16 of 19 up-regulated Ft genes in NH-JS1 were flowering repressors. In addition, 18 of 30 down-regulated Ft genes in NH-JS1 were flowering enhancers. This expression pattern was well correlated with the NH-JS1 phenotype. However, there were some exceptions. For example, the expression levels of *RsELF3* and *RsELF4*, both of which repress flowering, were reduced in NH-JS1. These two genes are components of the circadian clock (Covington et al., 2001; Doyle et al., 2002), suggesting that circadian rhythms may be partly responsible for the differences in Ft between the two lines. Alternatively, these specific circadian clock components may function differently than they do in *Arabidopsis*. This idea is supported by the observation that *RsELF3* was expressed 20-fold more highly in NH-JS2 than in NH-JS1. In *Arabidopsis*, ELF3 negatively regulates the expression of CO, FT, and GI (Kim et al., 2005). If *RsELF3* has similar functions in Ft control, then the expression level of *CO, FT*, and *GI* should be lower in NH-JS2 than in NH-JS1. However, *RsCO* and *RsGI* were more highly expressed in NH-JS2 than in NH-JS1, suggesting that RsELF3 may function differently than its *Arabidopsis* counterpart. Second, pathway analysis revealed that several pathways, including photoperiod and vernalization, were altered in NH-JS1. The expression pattern of genes in the vernalization pathway was well correlated with the NH-JS1 phenotype, suggesting that the radish vernalization pathway is similar to that of *Arabidopsis*. Interestingly, no autonomous pathway genes were found in the DEG list, even though 23 genes in this pathway were identified in radish (Table 3).

Regardless of vernalization treatment times, two repressor genes and 10 enhancer genes were up- and down-regulated in NH-JS1 vs. NH-JS2, respectively (Figure 3). In radish, these Ft genes are responsible for regulating bolting under vernalization. The expression profile of Ft genes exhibited that most of common Ft DEGs at any time of vernalization (above 12 genes) are overlapped (12 of 14 genes), indicating they expressed quantitatively and temperature-timely to develop different bolting time in the lines. Furthermore, pathway analyses of Ft DEGs suggested that the expression patterns of genes involved in the vernalization pathway perfectly matched the bolting characteristics of the two lines: seven repressors of the vernalization pathway were highly expressed, and five enhancers involved in the vernalization pathway were less expressed, in the late-bolting NH-JS1 line (Table 4). The 12 common Ft DEGs were biologically confirmed by qPCR analysis (Figures 4, 5).

### Possible divergence of flowering-time regulators between *Arabidopsis* and radish

FRI of *Arabidopsis* is a well-known repressor of Ft that positively regulates FLC expression (Choi et al., 2011). In our data set, we did not detect a radish FRI homolog. This may be because it is not abundantly expressed, or alternatively because there is simply no FRI homolog in radish. To resolve this issue, we performed BLAST analysis of the whole radish genome (Mun et al., 2015) using *Arabidopsis* FRI as a query sequence. However, we could not identify a candidate FRI ortholog with high homology and similar size to the *Arabidopsis* gene. One gene had 77% nucleotide sequence identity, but the region of homology spanned only half of the *Arabidopsis* FRI gene (data not shown). At present, we cannot exclude the possibility that this gene acts like *Arabidopsis* FRI, but it is also possible that yet another gene serves this function in radish. Recently, Nie et al. reported genes associated with bolting in radish based on *de novo* transcriptome analysis (Nie et al., 2015). They found one FRI gene in radish, but it is not clear whether its sequence is also present in the current version of the radish genome. Further research is required to resolve this discrepancy. A similar situation also arose regarding LFY, which is an integrator of Ft signaling (Weigel et al., 1992). LFY expression was not detected in our transcriptome data. As with FRI, this could have been either due to low expression or to the absence of such a gene in the genome. When we tried to find the gene using BLAST, we identified two homologous genes, but their cDNAs (5283 and 2490 bp) were much larger than that of *Arabidopsis* LFY (1263 bp). Most of the LFY sequence was present in the homologous region of the radish genes. Therefore, there may be some errors in gene prediction. Recently, Nie et al. reported three radish LFY genes (Nie et al., 2015). cDNA cloning and detailed analysis may help resolve the apparent discrepancy between our studies.

### A gene regulatory network for control of bolting time in radish

Three major flowering pathways, which differ in their response to vernalization treatment, have been defined, namely, vernalization, photoperiod, and gibberellin, based on Ft DEGs (Figure 6). Vernalization promotes flowering in response to a prolonged period of growth at low temperature, and we detected five Ft DEGs related to the vernalization pathway. *FLOWERING LOCUS C* (*FLC*, TBIU004737) is central to the vernalization process (Sung and Amasino, 2005). Two floral repressors, *RsFLC* and *RsMAF2*, an *RsFLC* homolog, were less expressed in the early-bolting NH-JS2, whereas three enhancers of the vernalization pathway were highly expressed in the early-bolting line. We propose that the vernalization pathway is closely associated with the difference in bolting time between the two inbred lines. Ft DEGs in the photoperiod pathway, *CCA1, LHY, ELF3*, and *GI*, play a role in facilitating the expression of *CONSTANS* as floral enhancers (Sawa et al., 2007; Imaizumi, 2010). Although *RsCO* was weakly expressed in the two lines, most genes related to the photoperiod pathway were highly expressed in the late-bolting line. Similar to the vernalization pathway, the photoperiod pathway showed coincidence with the bolting phenotype between the two lines. In *Arabidopsis*, the gibberellin pathway promotes flowering by up-regulating the *SOC1* genes (Bernier and Périlleux, 2005). GA hormone signaling occurs through proteolytic and non-proteolytic mechanisms when the GA receptor GID1 binds to GA (Murase et al., 2008). In proteolytic GA signaling, GID1 binds to negative regulators of GA responses called DELLA proteins, and the GID1-GA-DELLA complex is formed for destruction via the ubiquitin-proteasome pathway. Both radish DELLA protein and GA receptor genes, *RsGAI* and *RsGID1A*, were less expressed in the late-bolting line. However, further DEG analysis related to the GA pathway is needed to understand the relevancy to bolting time. Autonomous pathway-related genes were expressed at very low levels in all samples, suggesting that this pathway may not influence the difference in Ft between NH-JS1 and NH-JS2. These Ft DEGs from the flowering pathways converge on a key floral integrator, *RsSOC1*, and ultimately regulate *LFY* to determine the timing of the floral transition (Lee and Lee, 2010). However, we did not detect *RsLFY* in our transcriptomic analysis. Another floral integrator, *RsFT*, was also expressed at a low level, possibly because the samples we analyzed were derived from early-stage shoots. *RsSOC1* expression differed significantly between the two lines in a manner that depended on vernalization time, and its transcript levels may have an important impact on bolting in these two lines.

## Conclusions

This study demonstrates that differences in flowering traits between two inbred lines were consistent with the expression patterns of flowering-time genes involved in the vernalization pathway. In addition, our comparative transcriptome analysis elucidated the molecular basis of this divergence in bolting time. This is the first genome-wide comparative transcriptome analysis related to flowering traits in the radish.

## Author contributions

HC, YK conceived and designed the study and wrote the manuscript. WJ performed the data analysis and wrote the initial manuscript. HP, AL conducted bolting phenotyping and qPCR analysis and wrote the manuscript. SL contributed research materials.

### Conflict of interest statement

The authors declare that the research was conducted in the absence of any commercial or financial relationships that could be construed as a potential conflict of interest.

## Supplementary material

Figure S1 **Experimental design and data analysis**. Samples of the NH-JS1 and NH-JS2 inbred lines subjected to various vernalization treatments, used for RNA-Seq analysis. Ver, vernalization period; 1st and 2nd, independent biological replicates. The work-flow and algorithm used for detecting DEG and flowering-time genes are shown schematically.

Figure S2 **Pairs plot between the two replicate radish transcriptomes**.

Table S1 **Summary of RNA-Seq in two radish inbred lines**.

Table S2 **Results of read mapping in radish transcripts**.

Table S3 **Gene-specific primers used for qPCR analyses**.
