# Supplementary figures and images for: Identification of Flowering-Related Genes Responsible for Differences in Bolting Time between Two Radish Inbred Lines
Source: Front Plant Sci. 2016 Dec 9;7:1844. doi: 10.3389/fpls.2016.01844 (PMC5145866; doi:10.3389/fpls.2016.01844)

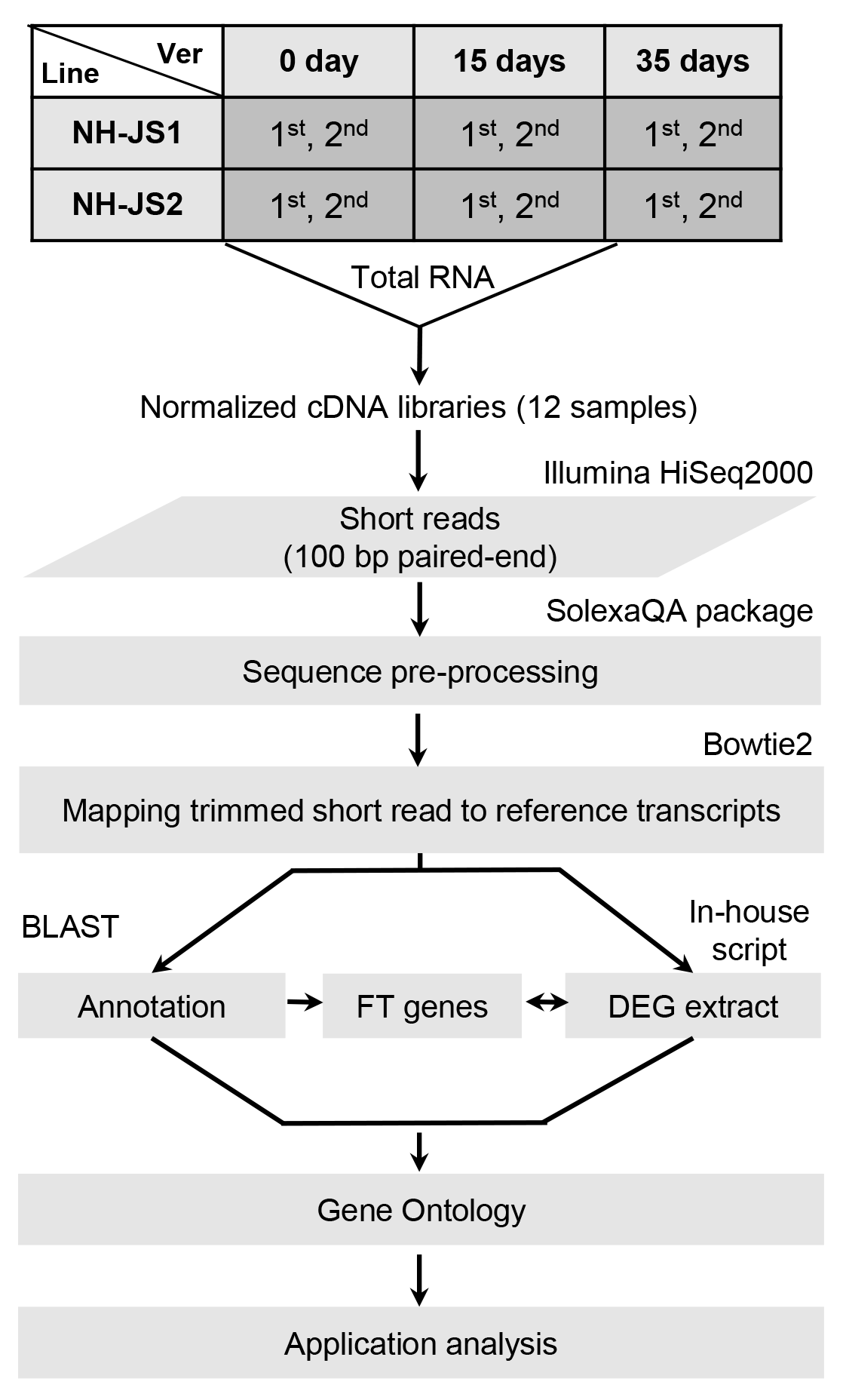

Supplement: Figure S1 — Experimental design and data analysis. Samples of the NH-JS1 and NH-JS2 inbred lines subjected to various vernalization treatments, used for RNA-Seq analysis. Ver, vernalization period; 1st and 2nd, independent biological replicates. The work-flow and algorithm used for detecting DEG and flowering-time genes are shown schematically. [file Image1.TIF]

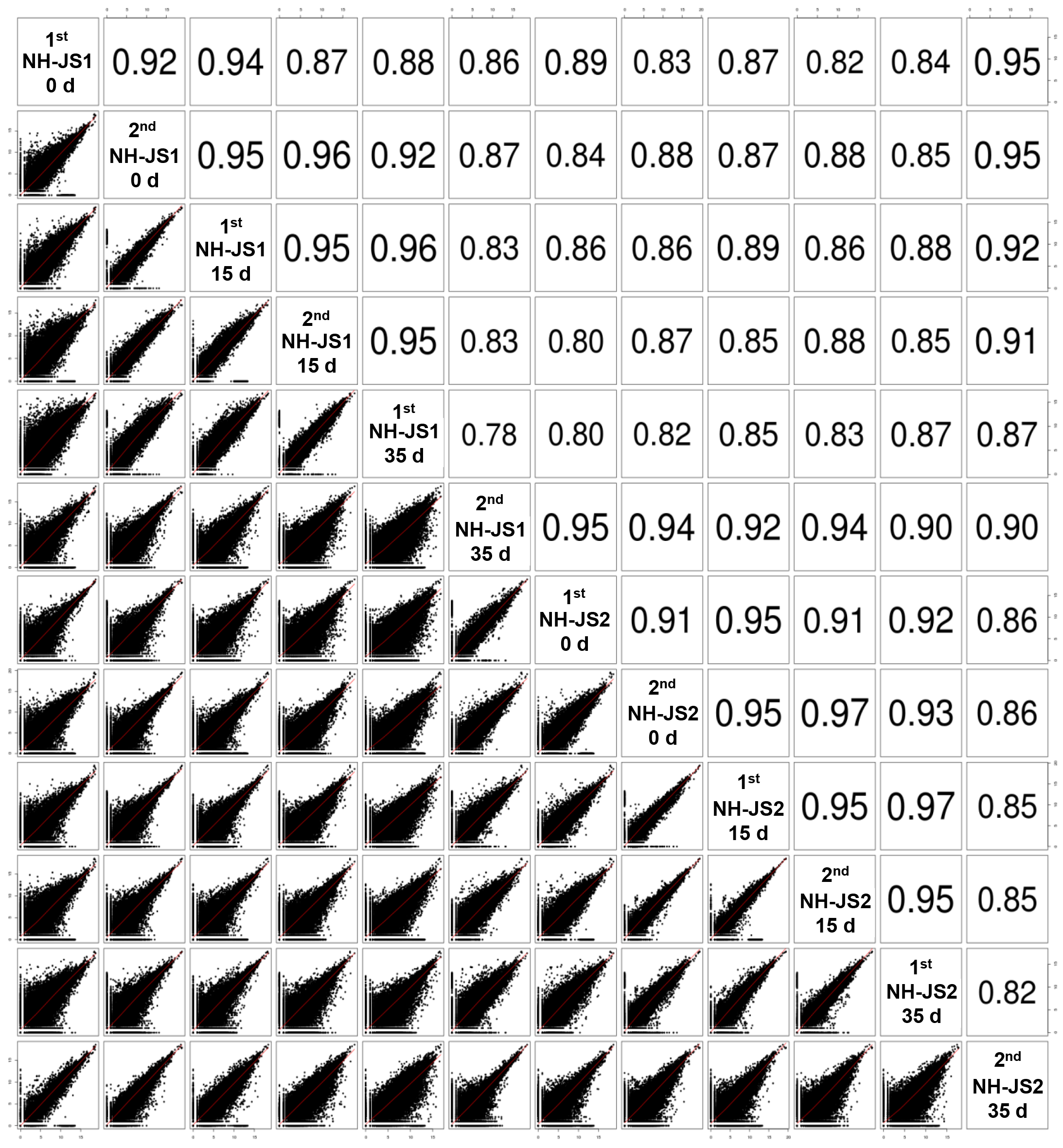

Supplement: Figure S2 — Pairs plot between the two replicate radish transcriptomes. [file Image2.TIF]
