# Supplementary material for: Identification of Flowering-Related Genes Responsible for Differences in Bolting Time between Two Radish Inbred Lines
Source: Front Plant Sci. 2016 Dec 9;7:1844. doi: 10.3389/fpls.2016.01844 (PMC5145866; doi:10.3389/fpls.2016.01844)
Supplement: Table S1 — Summary of RNA-Seq in two radish inbred lines. [file Table1.PDF]

Table S1. Summary of RNA-Seq in two radish inbred lines.

Sample description		Num. of reads	Total length (bp)	Avg. length	Trimmed/Raw (%)
1st	NH-JS1 0d.1	20,881,931	1,856,448,547	88.9	78.61%
	NH-JS1 0d.2	20,881,931	1,545,520,571	74.01	65.45%
	NH-JS1 15d.1	20,828,415	1,836,807,409	88.19	79.12%
	NH-JS1 15d.2	20,828,415	1,573,167,318	75.53	67.76%
	NH-JS1 35d.1	20,159,847	1,784,105,025	88.5	79.48%
	NH-JS1 35d.2	20,159,847	1,530,153,836	75.9	68.17%
	NH-JS2 0d.1	25,957,753	2,304,056,455	88.76	79.87%
	NH-JS2 0d.2	25,957,753	1,982,309,832	76.37	68.71%
	NH-JS2 15d.1	35,961,533	3,327,044,712	92.52	84.92%
	NH-JS2 15d.2	35,961,533	3,112,942,839	86.56	79.45%
	NH-JS2 35d.1	30,130,673	2,801,078,430	92.96	84.86%
	NH-JS2 35d.2	30,130,673	2,590,408,651	85.97	78.47%
2nd	NH-JS1 0d.1	21,577,164	2,050,796,889	95.04	82.24%
	NH-JS1 0d.2	21,577,164	1,833,405,889	84.97	73.52%
	NH-JS1 15d.1	23,225,001	2,214,303,036	95.34	83.01%
	NH-JS1 15d.2	23,225,001	1,987,138,447	85.56	74.50%
	NH-JS1 35d.1	21,343,009	2,007,645,968	94.07	85.75%
	NH-JS1 35d.2	21,343,009	1,907,306,196	89.36	81.47%
	NH-JS2 0d.1	25,780,686	2,416,946,972	93.75	85.71%
	NH-JS2 0d.2	25,780,686	2,308,863,986	89.56	81.88%
	NH-JS2 15d.1	23,525,676	2,216,964,361	94.24	84.89%
	NH-JS2 15d.2	23,525,676	2,084,214,708	88.59	79.81%
	NH-JS2 35d.1	24,187,970	2,271,846,057	93.92	86.16%
	NH-JS2 35d.2	24,187,970	2,170,896,079	89.75	82.33%
	**12 ea**	**587,119,316**	**51,714,372,213**	**87.85**	**79.01%**

· Trimmed/raw: Total length of trimmed read / total length of raw reads.

· Minimum length: If the read length is less than 25 bp it is excluded from the data.

· Num. of reads: Number of reads.

· Trimmed reads (Q > 20).

· Cleaned reads (minimum length > 25).
