# Supplementary material for: Identification of Flowering-Related Genes Responsible for Differences in Bolting Time between Two Radish Inbred Lines
Source: Front Plant Sci. 2016 Dec 9;7:1844. doi: 10.3389/fpls.2016.01844 (PMC5145866; doi:10.3389/fpls.2016.01844)
Supplement: Table S2 — Results of read mapping in radish transcripts. [file Table2.PDF]

Table S2. Results of reads mapping in radish transcripts.

Sample description		Total reads	Aligned 0 times		Aligned exactly 1 time		Aligned ≥ 1 times		Mapping rate	
			Reads (ea)	Raw (%)	Reads (ea)	Raw (%)	Reads (ea)	Raw (%)	Reads (ea)	Raw (%)
1st	NH-JS1 0d.1	20,881,931	1,065,489	5.10%	9,943,951	47.62%	9,872,491	47.28%	19,816,442	94.90%
	NH-JS1 0d.2	20,881,931	1,124,039	5.38%	10,203,711	48.86%	9,554,181	45.75%	19,757,892	94.62%
	NH-JS1 15d.1	20,828,415	1,036,803	4.98%	10,167,277	48.81%	9,624,335	46.21%	19,791,612	95.02%
	NH-JS1 15d.2	20,828,415	1,067,696	5.13%	10,373,440	49.80%	9,387,279	45.07%	19,760,719	94.87%
	NH-JS1 35d.1	20,159,847	1,135,331	5.63%	9,858,732	48.90%	9,165,784	45.47%	19,024,516	94.37%
	NH-JS1 35d.2	20,159,847	1,157,042	5.74%	10,018,848	49.70%	8,983,957	44.56%	19,002,805	94.26%
	NH-JS2 0d.1	25,957,753	1,345,016	5.18%	12,651,910	48.74%	11,960,827	46.08%	24,612,737	94.82%
	NH-JS2 0d.2	25,957,753	1,376,453	5.30%	12,919,021	49.77%	11,662,279	44.93%	24,581,300	94.70%
	NH-JS2 15d.1	35,961,533	1,977,042	5.50%	17,001,294	47.28%	16,983,197	47.23%	33,984,491	94.50%
	NH-JS2 15d.2	35,961,533	1,914,652	5.32%	17,138,811	47.66%	16,908,070	47.02%	34,046,881	94.68%
	NH-JS2 35d.1	30,130,673	1,785,362	5.93%	14,536,260	48.24%	13,809,051	45.83%	28,345,311	94.07%
	NH-JS2 35d.2	30,130,673	1,772,664	5.88%	14,655,639	48.64%	13,702,370	45.48%	28,358,009	94.12%
2nd	NH-JS1 0d.1	21,577,164	1,319,584	6.12%	10,320,728	47.83%	9,936,852	46.05%	20,257,580	93.88%
	NH-JS1 0d.2	21,577,164	1,308,537	6.06%	10,483,323	48.59%	9,785,304	45.35%	20,268,627	93.94%
	NH-JS1 15d.1	23,225,001	1,438,868	6.20%	11,407,662	49.12%	10,378,471	44.69%	21,786,133	93.80%
	NH-JS1 15d.2	23,225,001	1,434,612	6.18%	11,535,426	49.67%	10,254,963	44.15%	21,790,389	93.82%
	NH-JS1 35d.1	21,343,009	1,213,647	5.69%	10,360,422	48.54%	9,768,940	45.77%	20,129,362	94.31%
	NH-JS1 35d.2	21,343,009	1,198,821	5.62%	10,429,686	48.87%	9,714,502	45.52%	20,144,188	94.38%
	NH-JS2 0d.1	25,780,686	1,706,952	6.62%	12,251,684	47.52%	11,822,050	45.86%	24,073,734	93.38%
	NH-JS2 0d.2	25,780,686	1,703,649	6.61%	12,324,289	47.80%	11,752,748	45.59%	24,077,037	93.39%
	NH-JS2 15d.1	23,525,676	1,530,703	6.51%	11,166,606	47.47%	10,828,367	46.03%	21,994,973	93.49%
	NH-JS2 15d.2	23,525,676	1,503,043	6.39%	11,267,168	47.89%	10,755,465	45.72%	22,022,633	93.61%
	NH-JS2 35d.1	24,187,970	1,480,496	6.12%	11,505,948	47.57%	11,201,526	46.31%	22,707,474	93.88%
	NH-JS2 35d.2	24,187,970	1,479,575	6.12%	11,574,826	47.85%	11,133,569	46.03%	22,708,395	93.88%
	**12 ea**	**587,119,316**	**34,076,076**	**5.80%**	**284,096,662**	**48.45%**	**268,946,578**	**45.75%**	**553,043,240**	**94.20%**

· Aligned 0 times: The number of reads that do not mapping to the reference sequences.

· Aligned exactly 1 time: The number of reads that are map one times to the reference sequences.

· Aligned ≥1 times: The number of reads that map more than two times to the reference sequences.
