# Supplementary material for: Identification of Flowering-Related Genes Responsible for Differences in Bolting Time between Two Radish Inbred Lines
Source: Front Plant Sci. 2016 Dec 9;7:1844. doi: 10.3389/fpls.2016.01844 (PMC5145866; doi:10.3389/fpls.2016.01844)
Supplement: Table S3 — Gene-specific primers used for qPCR analyses. [file Table3.PDF]

Table S3 . Gene-specific primers used for qPCR analyses.

Name	Primer sequences		Annotation
	Forward [5'–3']	Reverse [5'–3']	
TBIU047119	TCGAATGGTGTAGTTCGAACGAGGAT	TCACTTCCACCGTTGTTCTTGGTCG	*B120*
TBIU057835	CCCACTCTCTCACACCCTCGTTTCA	GGTATGGATCCCAGCATCTTGCAGT	*DMC1*
TBIU039665	GGTGTACTCAGCCACGAGTTTAGCC	TGAAGCCACTGCTTCTGTTGACACG	*ABCC3*
TBIU054834	CCGACCCTAGAGCGAAACCGTCTT	TTCGGGAGTATGGTCCTCGCTTCC	*CRK2*
TBIU062462	TGCGGAAGCTTCTATGGAAGAGGA	AGGGAATTCTCCAGAAGGGAAGAGC	*MAP2A*
TBIU017689	CGACCGGAAAATCCTTGCAGCGTT	AGTGGTACGAACTCCTTTCGGCCA	*ELF3*
TBIU056908	ACCCGTAAAGCCGGTTAAAGCACG	AACGGTTCCGGCTTAACCTCTCCT	*CYP709B1*
TBIU057397	AGGAGACGATGATGACGACGACGA	ATAGCCGGATGGGACAAAGTTCGC	Serine/threonine-protein kinase
TBIU016676	GGAAGCCAGGGGGAACCTTTTTGC	GCCAACGTGCTCAACGGTCTACTC	Lipid-transfer protein
TBIU065173	GATCTTTTTAAGGCGAAAGCAGAG	AAGCATTAATGGAGGAAAGACCAG	*SOC1_1*
TBIU057467	CCTATGCCTTCTCCCAAGAGTTTA	GATCGTTATCTGAGGCATACCAAG	*SOC1_2*
RsFLC1	AGTAGCCGACAAGTTACCTTCTC	TAGCTCTTGTTACGGAGAGGGCG	*FLC*
RsCO1	CAGAGAAGAGAGCTGTGTTGGTTC	GGTGTATAGTCTCTTTGGGCGTCT	*CO*
RsVRN1	TGTTTGAAGACCTTGAAGACGAAG	AACTCTGAAGAAAGGGTTTGTTGG	*VRN1*
RsVRN2	CCATCGTTTCTTCCTAGATGCTT	TATCTGTGCCATTGGGTAAACAG	*VRN2*
RsFPA	AAGGGCTACAAGGGAGGTTATTC	TTTGACCTCATAGGGTTCCTCAT	*FPA*
RsLHY	CTCCTCTACTTTCCCCAATCTCA	GATCTTGCAGAGCTGTGTTCTGT	*LHY*
RsMAF2	CCAGGGTTTGGCTAGCCAGGT	ACACACGAGGTCTCTCAGCC	*MAF2*
RsSPA1	CTGATCCTCGACAACATCTGA	TCCTGAGGAATCTCCAGGAGC	*SPA1*
RsVIN3	GAAACCCGAGACCAATGTAGCA	AGACAGATCATCCATGAATGTCTC	*VIN3*
RsGID1A	ACAAGGGTAAGGGTTCTCAGG	TCTAAGCAGAGTCTACAGAC	*GID1A*
RsGAI	CGGTGGGATAGAGAAGGTTCTCGGGGTT	TCTGGTCTGGTCCGAACATATCTCATC	*GAI*
RsAGL19	CAGAAGTGAACACTGATGGCA	GAAGCCTAAGCTTAGAGATCTCCC	*AGL19*
RsNFYA4	GTCCGAGGGAAGAAGGTTAGA	ATTCTCCTTGTGGAATCAGTTGG	*NFYA4*
RsELF3[2]	AATCTGTATCGGTATCGACATC	TATCCTAACAGTGGTTGAGCTTG	*ELF3*
RsCCA1	GCTCAATGGAAGCCAAAGAGAGT	GTTACAGGAACACTATGGACATG	*CCA1*
RsGI	GCACTGTGCAATGTGGTATCT	CCATCAGTTGCCCTGAGAAGAA	*GI*
RsACT1	TACCGCAA AGAGCAGTTCGTCAGTG	GAGCGATGGCTGGAACAGTACTTCAG	*Actin*
